# Evaluating volleyball interventions for enhancing physical fitness in healthy individuals: a systematic review and meta-analysis

**DOI:** 10.3389/fspor.2026.1800110

**Published:** 2026-05-12

**Authors:** Yimou Mao, Kim Geok Soh, Siswantoyo, Xinzhi Wang

**Affiliations:** 1 Faculty of Educational Studies, Department of Sports Studies, Universiti Putra Malaysia, Serdang, Selangor, Malaysia; 2 Faculty of Sport Sciences, Universitas Negeri Yogyakarta, Yogyakarta, Indonesia

**Keywords:** healthy individuals, meta-analysis, physical fitness, systematic review, training intervention, volleyball

## Abstract

**Background:**

Volleyball, which combines aerobic and anaerobic activities, has been suggested to improve various Physical Fitness (PF) components. However, the specific effects of volleyball-based interventions on fitness among healthy individuals and across different durations have yet to be fully summarised.

**Objective:**

This study aimed to compile and analyse data from randomised controlled trials (RCTs) and other controlled studies to assess the effects of volleyball interventions on PF.

**Methods:**

A systematic search was conducted in PubMed, EBSCOhost (SPORTDiscus), Web of Science, Scopus, and the Cochrane Library to identify RCTs and controlled studies examining the effects of volleyball interventions on PF. Eligible studies reported at least one PF outcome. Risk of bias was assessed using the Cochrane Risk of Bias tool. Heterogeneity was evaluated using the I² statistic.

**Results:**

Twelve RCTs, including 656 participants, were incorporated into this meta-analysis, which showed that volleyball interventions significantly improved muscular endurance (SMD = 1.16, 95% CI: 0.44–1.89), cardiovascular endurance (SMD = 0.72, 95% CI: 0.40–1.05), muscular strength (SMD = 0.20, 95% CI: 0.04–0.36), flexibility (SMD = 0.37, 95% CI: 0.17–0.57), agility (SMD = −0.75, 95% CI: −0.99 to −0.51), speed (SMD = −0.32, 95% CI: −0.55 to −0.10), and balance (SMD = −0.98, 95% CI: −1.90 to −0.05).

**Conclusion:**

This study demonstrates that volleyball interventions lead to statistically significant improvements in multiple components of PF. These findings support the use of volleyball interventions as an effective movement-training modality for improving PF and performance in healthy populations.

**Systematic Review Registration:**

https://crd.york.ac.uk/PROSPERO/view/CRD42024537624, PROSPERO CRD42024537624.

## 1 Introduction

Regular physical activity at all ages, from childhood to old age, is essential for maintaining and improving physical fitness (PF) (1, 2). Elevated PF levels are associated with numerous health benefits, including a reduced risk of chronic diseases, improved mental health, and enhanced quality of life (3, 4). PF components can be divided into two groups (5): those related to health and those related to skills more relevant to athletic ability (6–8). Health-related fitness generally encompasses body composition, cardiovascular endurance, flexibility, muscular endurance, and strength (9, 10). The other skill-related fitness components encompass speed, balance, power, agility, reaction time, and coordination (9–11).

Volleyball is characterized by intermittent high-intensity activity alternating with lower-intensity periods (12, 13). Volleyball training combines repeated bursts of anaerobic activity, such as jumping, spiking, and rapid directional changes, with aerobic endurance demands during prolonged rallies and continuous play (14, 15). These features require volleyball players to perform specific technical actions, including repeated jumping and landing, blocking, overhead passing, and coordinated whole-body movements. These movement characteristics not only reflect the sport's high technical demands but may also contribute to improvements in lower-limb power, dynamic balance, agility, coordination, and neuromuscular control (14, 15). Taken together, the physical and technical characteristics of volleyball require players to possess rapid adjustments in movement and dynamic postural control, which have been shown to improve agility, balance, muscular power, and cardiovascular endurance (14–16). Beyond these general training benefits, volleyball, compared with contact-oriented team sports such as football and basketball, is a net-separated sport with relatively less direct bodily collision. This characteristic may make it a safer and more practical option in school, community, and recreational settings (16–18).

Interventions in volleyball have been demonstrated to improve physical fitness in specific studies. For example, a 10-week study involving 24 men aged 35–55 who participated in volleyball sessions twice to three times per week for 90 min each showed a significant improvement in cardiovascular endurance (16). Similarly, the intervention group in a volleyball study by Hasan Sozen (17) showed substantial improvements in sit-ups, a 10 × 5-metre round-trip run, seated forward bends, and the flamingo balance test. Current experiments have validated the effects of volleyball interventions on physical health. However, the specific effects have yet to be fully summarised.

While several studies have demonstrated the positive effects of volleyball interventions on PF (11, 18–20), these studies often differ in sample sizes, intervention durations, and outcome measures, leaving gaps in the overall understanding of volleyball's effectiveness. There are still gaps in the existing literature, particularly regarding the need for single-intervention duration (SMD), intervention duration (ID), and frequency across different age groups. Therefore, this meta-analysis aimed to systematically evaluate the available research on the impact of volleyball interventions on key physical fitness components, including cardiovascular endurance, muscular strength, flexibility, agility, speed, balance, body composition, power, and muscular endurance. By focusing solely on studies involving healthy individuals, this review aims to provide a clearer understanding of the specific benefits of volleyball interventions for enhancing physical fitness in healthy populations and to identify potential areas for future research. Meta-analysis, a statistical tool that contributes to evidence-based practice, provides pooled effect estimates from individual studies in systematic reviews (21–23). Meta-analysis enables researchers to assess the effectiveness of interventions across various settings and populations (24). For volleyball interventions, meta-analyses allow the identification of overall trends and effect sizes.

## 2 Materials and methods

### 2.1 Study design

This review and meta-analysis adhered to PRISMA (Preferred Reporting Items for Systematic Reviews and Meta-Analyses) guidelines to promote standardization and transparency throughout the process (25, 26). The research in this paper has been registered in the PROSPERO database under registration number CRD42024537624 to ensure the openness and replicability of the methodology and to increase the credibility and scientific rigor of the study (25, 27).

### 2.2 Search strategy

A systematic review and meta-analysis were conducted to identify studies evaluating the effects of volleyball interventions on PF, using multiple databases. The screening and inclusion of studies are detailed below.

#### 2.2.1 Literature search

The keyword search for this study was about volleyball and PF. The combination of search terms included (“Volleyball*” OR “volleyball training” OR “volleyball intervention*” OR “volleyball teaching”) AND (“Physical Fitness” OR “Cardiovascular endurance” OR “Agility” OR “Body composition” OR “Power” OR “Flexibility” OR “Balance” OR “Muscular strength” OR “Speed” OR “Muscular endurance”). Databases used include Cochrane Library, Web of Science, EBSCOhost (SPORTDiscus), Scopus, and PubMed. The search was conducted for articles published from the earliest available date in the databases through May 28, 2025, ensuring inclusion of the most recent and relevant studies within the scope of this research.

### 2.3 Inclusion criteria

Inclusion criteria in this research were structured according to the PICOS framework (28). (P) Population: Healthy individuals of all genders, including preschool-aged children, adolescents, and adults. (I) Intervention: Volleyball interventions encompass volleyball training (VT), modified recreational volleyball (MRV), volleyball skill-based training (VSBT), and teaching games for understanding (TGfU). (C) Control group (CG): No intervention, other sports, or regular basic exercises, including regular recreational volleyball (RRV). (O) Outcomes: PF measures muscular strength, flexibility, speed, body composition, balance, cardiovascular endurance, agility, and muscular endurance (29–31). (S) Study type: Randomised controlled trials (RCTs) and controlled trials. These criteria were designed to identify relevant studies and provide sufficient data on the effects of volleyball interventions on physical fitness (PF).

### 2.4 Exclusion criteria

This systematic review and meta-analysis excluded studies according to the following criteria:

Population: Studies involving specific health conditions, injuries, or disabilities.
Interventions: Studies whose primary intervention was not volleyball training or volleyball-related physical activity. For example, studies focusing on other types of physical activity or non-physical interventions.
(3) control group: Studies without a CG, or those with a CG that did not allow for a meaningful comparison of intervention effects, were excluded.
Study types: non-experimental studies, reviews, opinion articles, case reports, cross-sectional studies, meta-analyses, and theoretical papers.
Outcomes: Studies that did not report on at least one of the specified measures of PF: cardiovascular endurance, muscular strength, flexibility, agility, speed, body composition, power, balance, or muscular endurance.

By applying these exclusion criteria, only studies of strong relevance and quality were selected, thereby facilitating a focused and accurate analysis of the effects of volleyball interventions on PF.

### 2.5 Study selection

The study selection process involved several essential steps to ensure only high-quality, eligible studies were included in this systematic review and meta-analysis. We first used Zotero, a reference management software, to efficiently screen and exclude duplicate literature (32–34). Two researchers independently screened titles to remove duplicates, non-RCT studies, review literature, and conference papers. Next, they reviewed the abstracts to exclude studies that did not meet the inclusion criteria for this paper (35). If disagreements arose during the screening process, the researchers resolved them through discussion. If the discussion did not result in agreement, the discussion was handled with the assistance of a third researcher, who provided input to ensure accuracy and fairness in the screening process (36). Eligible literature was thoroughly reviewed, and studies that did not meet the criteria were ultimately excluded to ensure the included studies were highly relevant and of high quality (37).

### 2.6 Data extraction and quality assessment

Two reviewers independently extracted data from the included studies, including the first author and year of publication, country, population, age (mean ± SD), total sample size, intervention, control condition, study design, and outcome measures. Any disagreements were resolved through discussion, and where necessary, a third reviewer was consulted. Two researchers independently assessed the risk of bias in the included studies according to study design. For randomised controlled trials, the latest version of the Cochrane risk-of-bias tool (RoB 2) was used, covering five domains: bias arising from the randomisation process, bias due to deviations from intended interventions, bias due to missing outcome data, bias in measurement of the outcome, and bias in selection of the reported result (38–40). For non-randomised studies of interventions, the ROBINS-I tool was used, covering seven domains: bias due to confounding, bias in the selection of participants into the study, bias in the classification of interventions, bias due to deviations from intended interventions, bias due to missing data, bias in measurement of outcomes, and bias in selection of the reported result (41–43).

### 2.7 Data analysis

All continuous variables are presented as means ± SD for studies that used volleyball as the intervention. Continuous outcomes are reported as MD or SMD, along with 95% confidence intervals (CI) (44–47). MD is computed using a consistent scale to represent the absolute difference between the group means (experimental and control). SMD: SMD combines data from trials that use different scales (47–49). A random-effects model is applied when heterogeneity (*I*²) exceeds 50%, whereas a fixed-effects model is used if *I*² is 50% or below (50) to account for variability across studies. This model accounts for variability across studies and yields more generalisable results. The funnel plot was used to visually assess asymmetry, which may indicate publication bias (51). Begg's test will detect bias statistically, with a *p*-value < 0.05 indicating significant bias (52). Similarly, Egger's test will detect bias, where a *p*-value less than 0.05 suggests substantial bias (52). These methods, implemented in Stata, help ensure the robustness and reliability of the results.

## 3 Results

### 3.1 Literature screening

**Figure 1:**
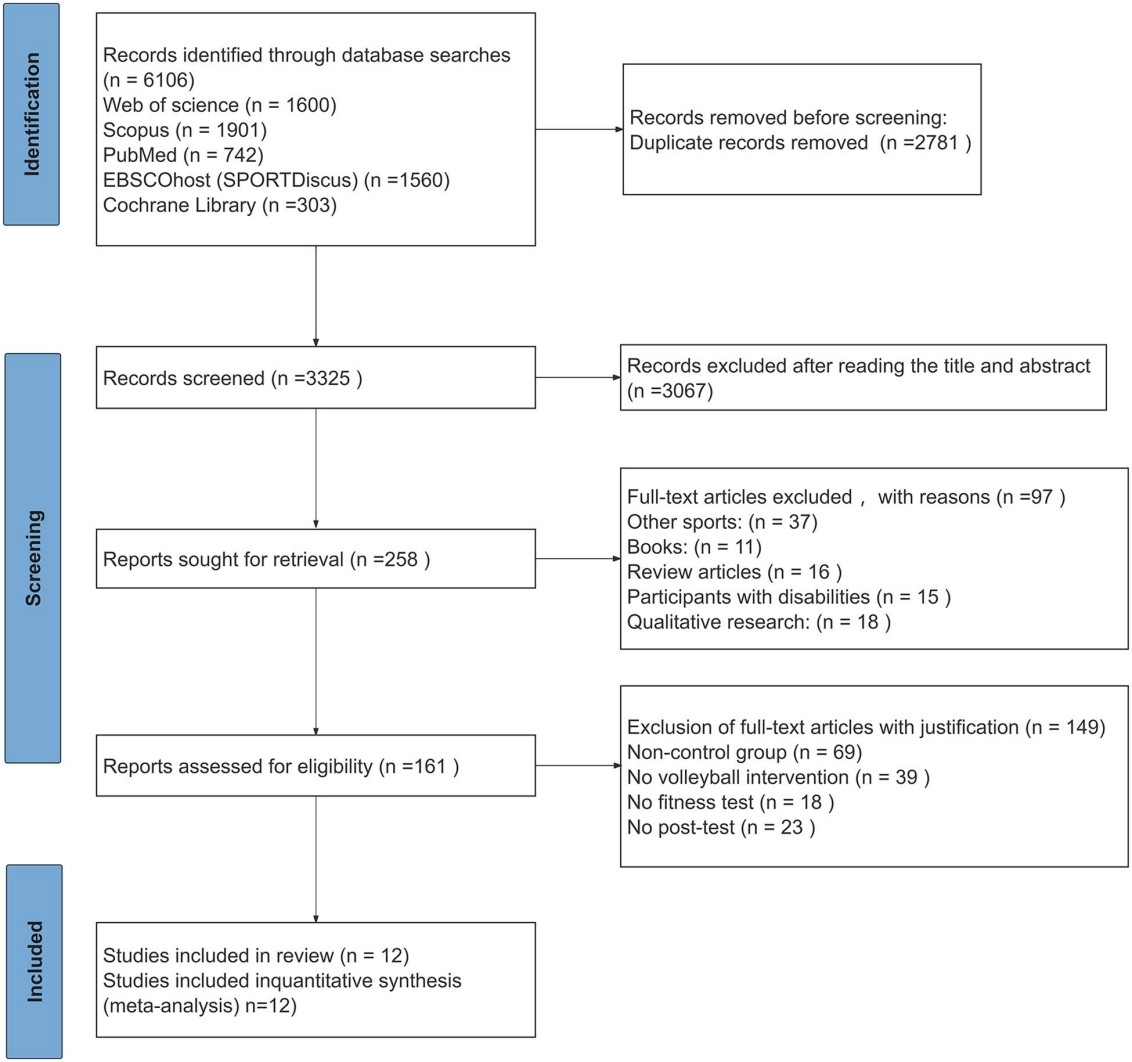
PRISMA flow diagram.

A comprehensive database search identified 6,106 articles from the following sources: Web of Science, Scopus, PubMed, EBSCOhost (SPORTDiscus), and the Cochrane Library. After removing 2,781 duplicates, the remaining 3,325 documents entered the screening stage. The process began with the exclusion of 3,067 articles that, based on their titles and abstracts, did not meet the inclusion criteria. Subsequently, 258 reports underwent full-text assessment. Of these, 161 articles met the inclusion criteria. However, during further evaluation, we excluded 149 full papers for the following reasons: no control group: 69, no volleyball intervention: 39, no fitness test: 18, and no post-test: 23. Ultimately, 12 studies satisfied the inclusion criteria and were integrated into this systematic review and meta-analysis, the PRISMA flow diagram is shown in (Figure 1).

### 3.2 Quality assessment of the included studies

**Figure 2:**
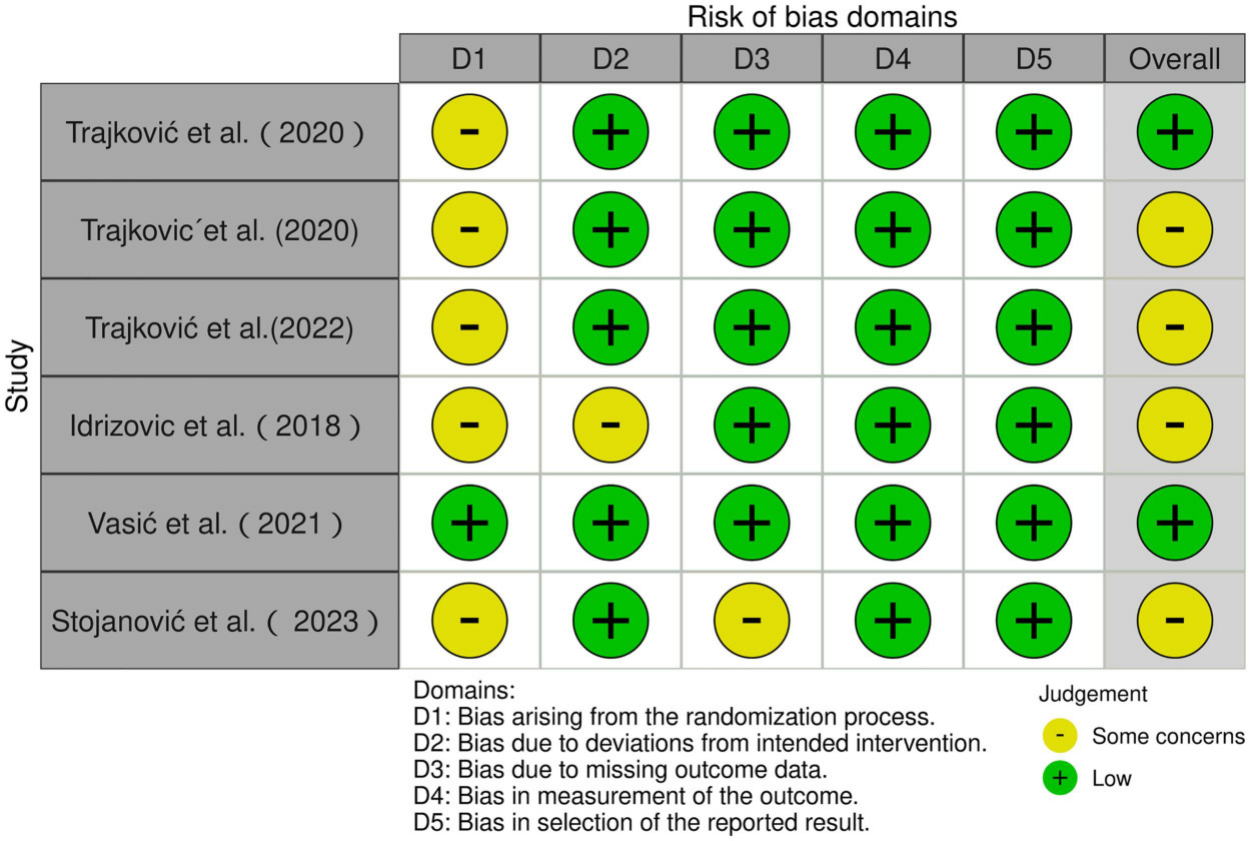
RoB-2 assessments.

The risk of bias of six cluster-randomised controlled trials was assessed using the RoB 2 tool (16, 20, 55, 56, 58, 61), and that of six non-randomised controlled trials was assessed using the ROBINS-I tool (17, 53, 54, 57, 59, 60). As shown in Figure 2, among the six cluster-randomised controlled trials, two studies were judged to be at low overall risk of bias (16, 20), while four were judged to raise some concerns (55, 56, 58, 61). Five studies were judged as raising some concerns in the domain of the randomisation process (16, 20, 55, 58, 61), mainly because they reported that random allocation had been used but did not provide sufficient details of the randomisation procedure. The remaining study was judged to be at low risk in this domain (56). In addition, one study raised some concerns regarding deviations from intended interventions (58), and one study raised some concerns regarding missing outcome data (61), whereas the other domains were generally judged to be at low risk.

**Figure 3:**
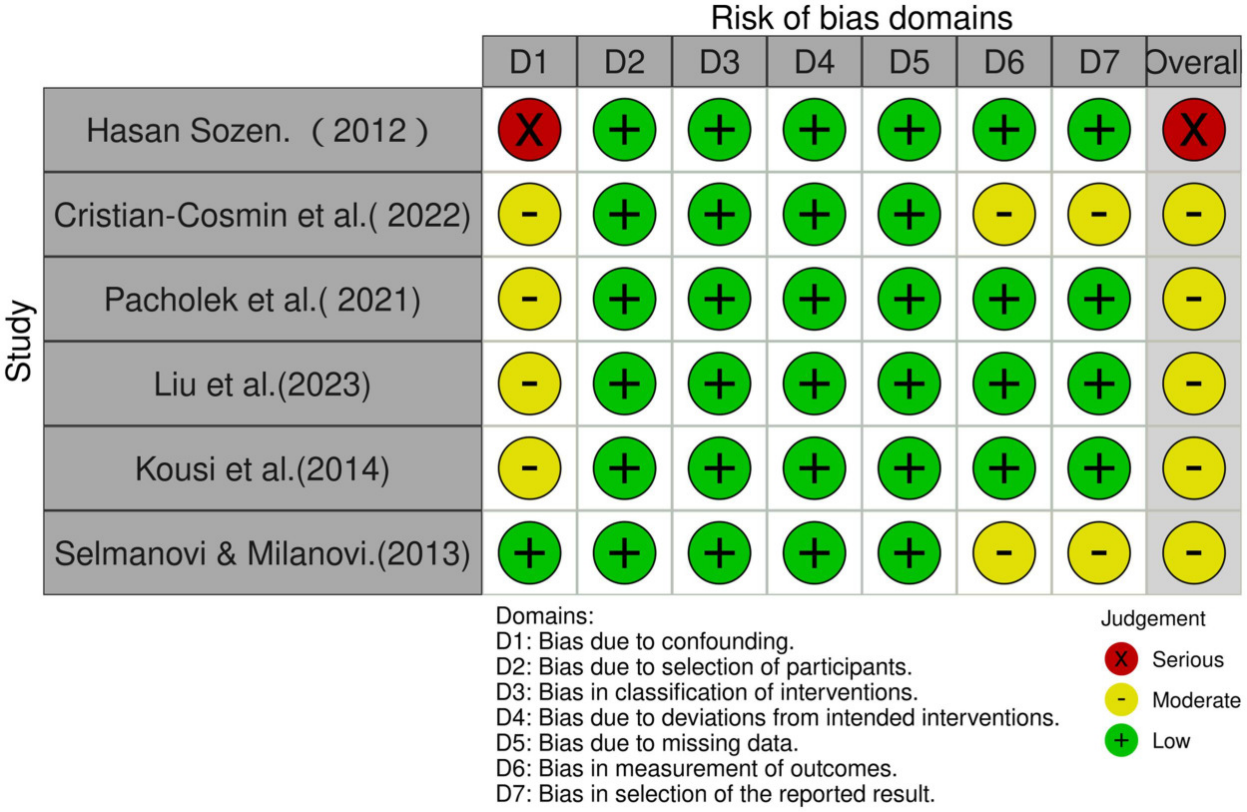
ROBINS-I assessments.

As shown in Figure 3, among the six non-randomised controlled trials, one study was judged to be at serious risk of bias (17), four at moderate risk (53, 54, 59, 60), and one at low risk (57). The serious overall risk of bias identified in study (17) was mainly attributable to confounding. The four studies judged to be at moderate risk of bias (53, 54, 59, 60) were likewise affected, primarily due to confounding, while some also showed limitations in outcome measurement or in the selection of the reported results (53, 60).

As shown in Figure 3, one of the six non-randomised controlled trials was judged to be at serious risk of bias (17), whereas the other five were judged to be at moderate risk of bias (53, 54, 57, 59, 60). The serious rating for the study (17) was mainly due to confounding arising from comparing school volleyball team members with sedentary students from different schools, without random allocation. Among the studies rated as moderate risk, confounding was the main concern in studies (53, 54, 57, 59) largely because group allocation depended on availability, pre-existing class assignment, or prior training status rather than fully controlled allocation procedures. By contrast (60) the moderate rating for study (60) was mainly related to outcome measurement and selection of the reported result, as assessor blinding and a pre-specified analysis plan were not described.

### 3.3 Characteristics of included studies

This review included 12 RCTs that examined diverse populations across various countries, including overweight children, untrained adolescents, and adults. Interventions in the Experimental Group (EG) included multiple types of volleyball training programs with varying details, including sessions held 1 to 4 times per week and durations of 4 weeks to 9 months. Outcome indicators assessed in these studies included muscular endurance, agility, strength, flexibility, balance, speed, power, cardiovascular endurance, and body composition (as measured by body mass index). The interventions in the EG outcome measures assessed across these studies included muscular endurance (5 studies) (17, 53, 54, 57, 60), agility (5 studies) (17, 53, 54, 60, 61), muscular strength (11 studies) (16, 17, 20, 53, 54, 56–61), balance (3 studies) (17, 53, 60), Speed (5 studies) (17, 53, 57, 58, 60), body composition (6 studies) (16, 54–56, 59, 61), flexibility (6 studies) (17, 54, 57, 58, 60, 61), power (5 studies) (20, 58–61), and cardiovascular endurance (5 studies) (16, 20, 56, 57, 61). A summary of the characteristics of the included studies is presented in Table 1.

**Table 1:** Descriptive characteristics of the included studies.

Author, years	Country	Population	Age (mean + SD)	Total/male/female	Intervention	Control	Study design	Outcome
(Hasan Sozen, 2012) (17)	Turkey	High school students	EG:Female15.28 ± 46 Male 15.7 ± 0.77	EG:31/17/14	VT SID: not mentioned Freq: not mentioned Duration: One semester	CON	QE	ME:Sit-ups A:5 m SR MS:SBJ F: SAR B: FBT S: PTT
			CG:Female15.28 ± 46 Male 15.35 ± 0.60	EG:31/17/14				
(Selmanovi & Milanovi, 2013) (60)	Croatia	Fifth grade elementary school students	EG:11 years (± 6 months)	EG:45/45/0	VT SID: 45 min Freq: 1 time a week Duration: 9-month	CON	QE	ME:Sit-ups A:5 m SR MS:SBJ F: MPRR B: Low beam stand P: MBT S: 20-m sprint
			CG:11 years (± 6 months)	CG:42/42/0				
(Kousi et al., 2014) (59)	Greece	Prepubescent boys	EG:10.5 ± 0.9	EG:15/15/0	VB training SID: NA Freq: 3 times a week Duration: School/competitive season 2011–2012	CON	QE	MS:CMJ P: SJ BC: BMI
			CG:10.1 ± 0.7	CG:15/15/0				
(Idrizovic et al., 2018) (58)	Montenegro	Junior female volleyball players	EG:16.6 ± 0.6	EG:17/0/17	VSBT SID: 40–60 min Freq: 2 times a week Duration (Period): 12 weeks	RRV	RCT	MS:CMJ F: SAR P: MBT S: 20-m sprint
			CG:16.6 ± 0.6	CG:17/0/17				
(Trajković et al., 2020) (16)	Serbia	Healthy untrained men	EG:44.7 ± 6.34	EG:12/12/0	VT SID: 90 min Freq: 2–3 times a week Duration: 10 weeks	CON	RCT	CE:Yo-Yo IR Test MS:Handgrip BC: BMI
			CG:42.9 ± 8.72	CG:12/12/0				
(Trajkovićet al., 2020) (20)	Serbia	Adolescents (high school students)	EG:15.5 ± 0.7	EG:56/38/17	VT SID: 45 min Freq: 2 times a week Duration: 8-month	CON	RCT	CE: Yo-Yo IR Test MS:CMJ P: MBT
			CG:15.7 ± 0.6	CG:51/35/19				
(Vasić et al., 2021) (56)	Serbia	Healthy untrained men	EG:43.5 ± 5.3	EG:17/17/0	MRV SID: 70 min Freq: 2 times a week Duration: 12 weeks	RRV	RCT	CE:Yo-Yo IR Test MS:Handgrip BC: BMI
			CG:41.9 ± 5.7	CG:17/17/0				
(Trajković et al., 2022) (55)	Serbia	Overweight adolescent girls	EG:15.6 ± 0.5	EG:22/0/22	VT SID: 90 min Freq: 2 times a week Duration: 12 weeks	CON	RCT	BC: BMI
			CG:15.5 ± 0.7	CG:20/0/20				
(Pacholek et al., 2021) (54)	Saudi Arabia	Male university students	EG:20.2 ± 1.2	EG:14/14/0	VT SID: 50 min Freq: 4 times a week Duration: 4 weeks	CON	QE	ME:Sit-ups A:5 m SR MS:SBJ F: SAR BC: BMI
			CG:20.5 ± 1.5	CG:14/14/0				
(Cristian-Cosmin et al., 2022) (53)	Romania	Overweight and obese children	EG:9.4 ± 1 years old	EG:14/0/14	VT SID: 90 min Freq: 3 times a week Duration: 6 months	CON	QE	ME:Sit-ups A: 5 m SR MS:SBJ B: FBT S: PTT
			CG:9.1 ± 0.9 years old	CG:14/0/14				
(Stojanović et al., 2023) (61)	Serbia	Primary school students	EG:13.3 ± 0.3	EG:39/20/19	VT SID: 45 min Freq: 1 time a week Duration: 12 weeks	CON	CRCT	CE: Maximal Oxygen Uptake VO2max MS: CMJ F: SAR P: SJ A: Agility T-test (s) BC: BMI
			CG:13.3 ± 0.3	CG:49/25/24				
(Liu et al., 2023) (57)	China	College students	EG:Not explicitly mentioned	EG:50/25/25	TGFU VB training SID: 45 min Freq: 1 time a week Duration: 1 semester	RRV	QE	CE:1,000 m,800m ME: Sit-ups MS: SBJ F: SAR S: 50m-run
			CG:Not explicitly mentioned	CG:50/25/25				

EG, experimental group; CG, control group; VT, volleyball training; SID, single intervention duration; NA, not Available; CON, control group receiving regular exercise (no sport); RCV, regular recreational volleyball; TGfU, teaching games for understanding; CE, cardiovascular endurance; ME, muscular endurance; MS, muscular strength; A, agility; F, flexibility; BC, body composition; P, power; B, balance; S, speed; VB, volleyball; 5 m SR, 10 × 5 m shuttle-run; SBJ, standing broad jump; SAR, sit-and-reach; CMJ, countermovement jump; MPRR, wide leg forward bend; Yo-Yo IR Test, Yo-Yo Intermittent Recovery Test; MBT, medicine ball toss; SJ, squat jump; FBT, Flamingo Balance Test; PTT, plate tapping test; RCT: randomised controlled trial; CRCT, cluster-randomised controlled trial; QE, quasi-experimenta.

### 3.4 Meta-analysis results

#### 3.4.1 The impact of volleyball interventions on muscular endurance

**Figure 4:**
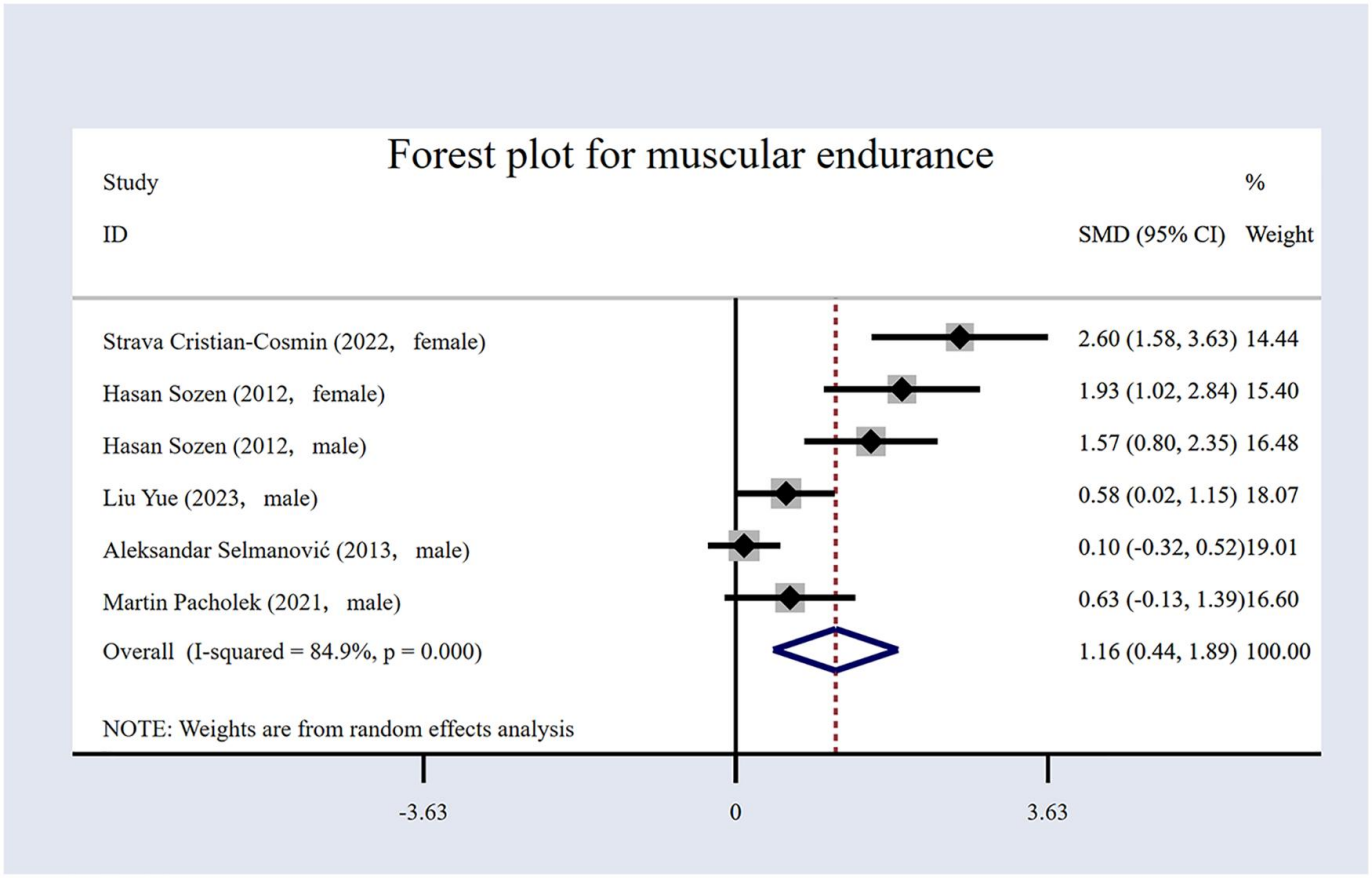
Forest plot for muscular endurance.

The five included RCTs were examine (17, 53, 54, 57, 60), with 255 participants (Table 1), of whom 129 were in the EG and 126 in the CG. The forest plot for muscular endurance (Figure 4) indicates that the volleyball intervention significantly enhanced participants’ muscular endurance. The intervention resulted in an *SMD* of 1.16 [95% (CI): 0.44 to 1.89, *p* = 0.002]. However, a high degree of heterogeneity was observed among the studies (*I*² = 84.9%, *p* < 0.001), indicating variability in the effects across studies.

#### 3.4.2 The impact of volleyball interventions on cardiovascular endurance

**Figure 5:**
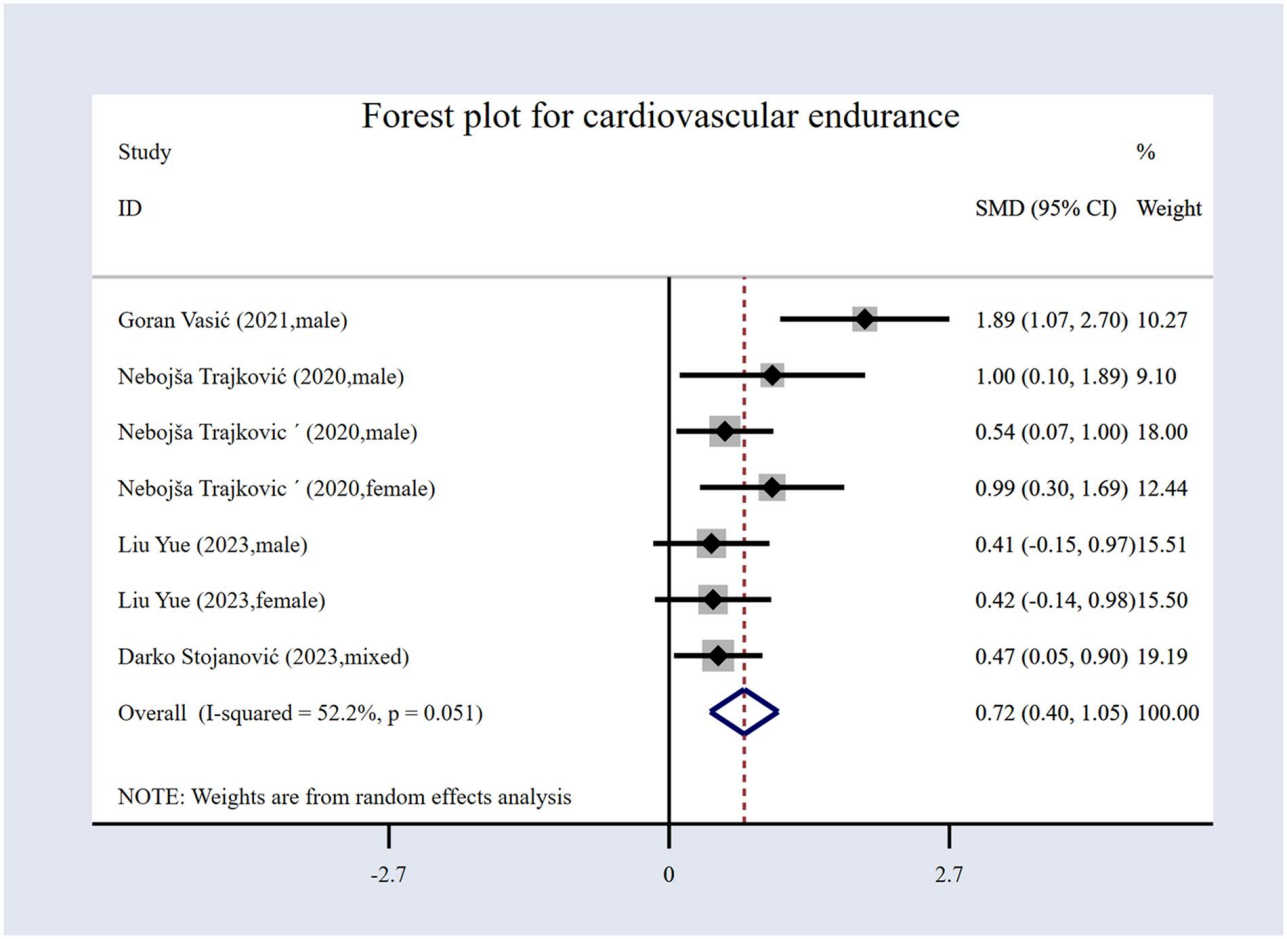
Forest plot for cardiovascular endurance.

Five studies were included in this analysis (16, 20, 56, 57, 61); the study comprised 353 participants overall, as shown in Table 1, with 174 in the EG and 179 in the CG. The forest plot for cardiovascular endurance (Figure 5) illustrates the effects of volleyball interventions. The results indicate that volleyball interventions significantly improved cardiovascular endurance, with an *SMD* of 0.72 [95% (CI): 0.40 to 1.05, *p* < 0.001]. The studies showed moderate heterogeneity, with an *I*² value of 52.2% (*p* = 0.051). This suggests that while there was some variability in the results, the overall effect was consistent and statistically significant.

#### 3.4.3 The impact of volleyball interventions on muscular strength

**Figure 6:**
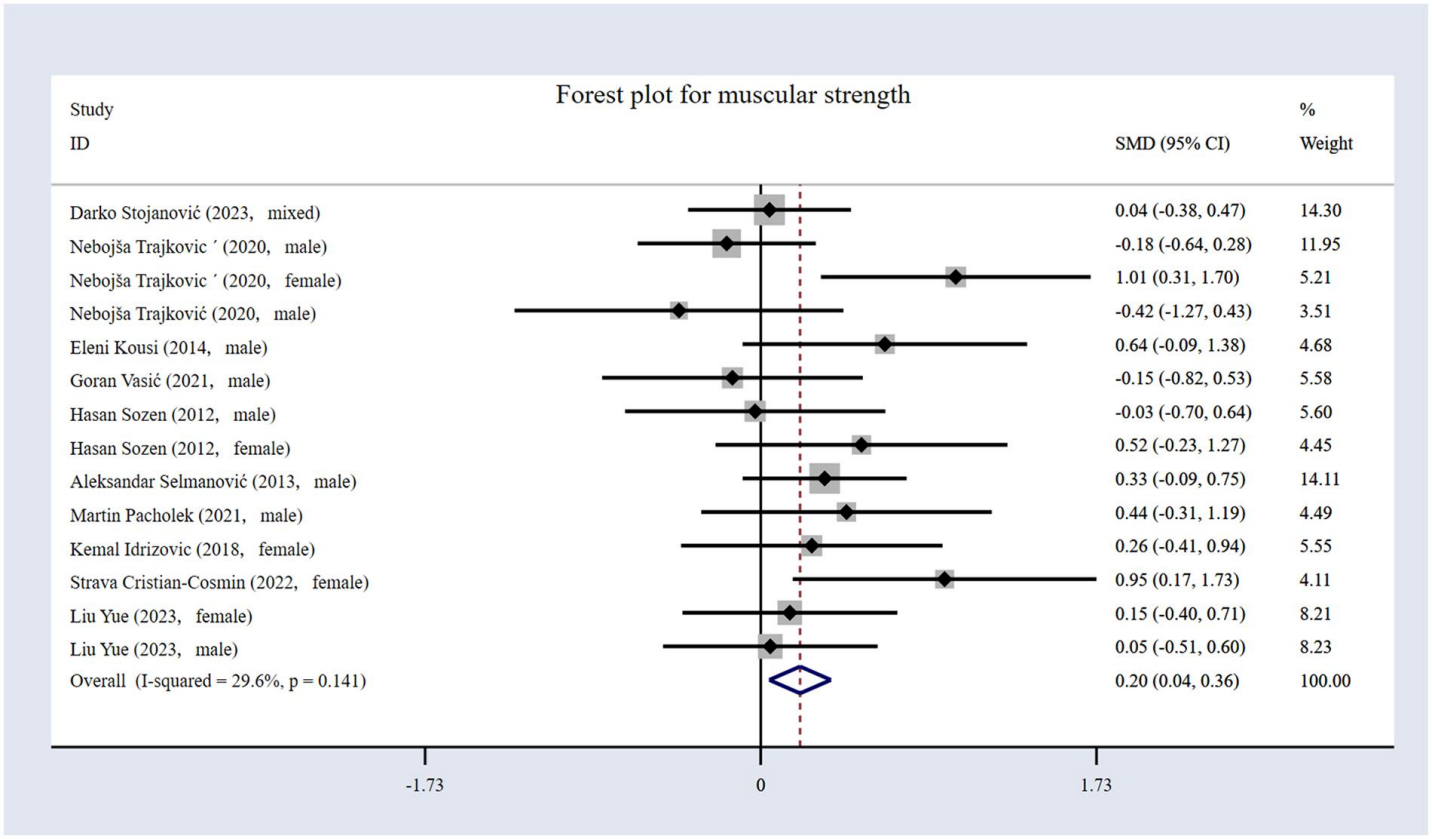
Forest plot for muscular strength.

The eleven included studies (16, 17, 20, 53, 54, 56–61) comprised 623 participants overall, as shown in Table 1, with 307 in the EG and 316 in the CG. The forest plot for muscular strength (Figure 6) illustrates that the results indicate that volleyball interventions significantly improved muscular strength, with an *SMD* of 0.20 [95% (CI): 0.04 to 0.36, *p* = 0.012]. The heterogeneity was moderate (*I*² = 29.6%, *p* = 0.141).

#### 3.4.4 The impact of volleyball interventions on flexibility

**Figure 7:**
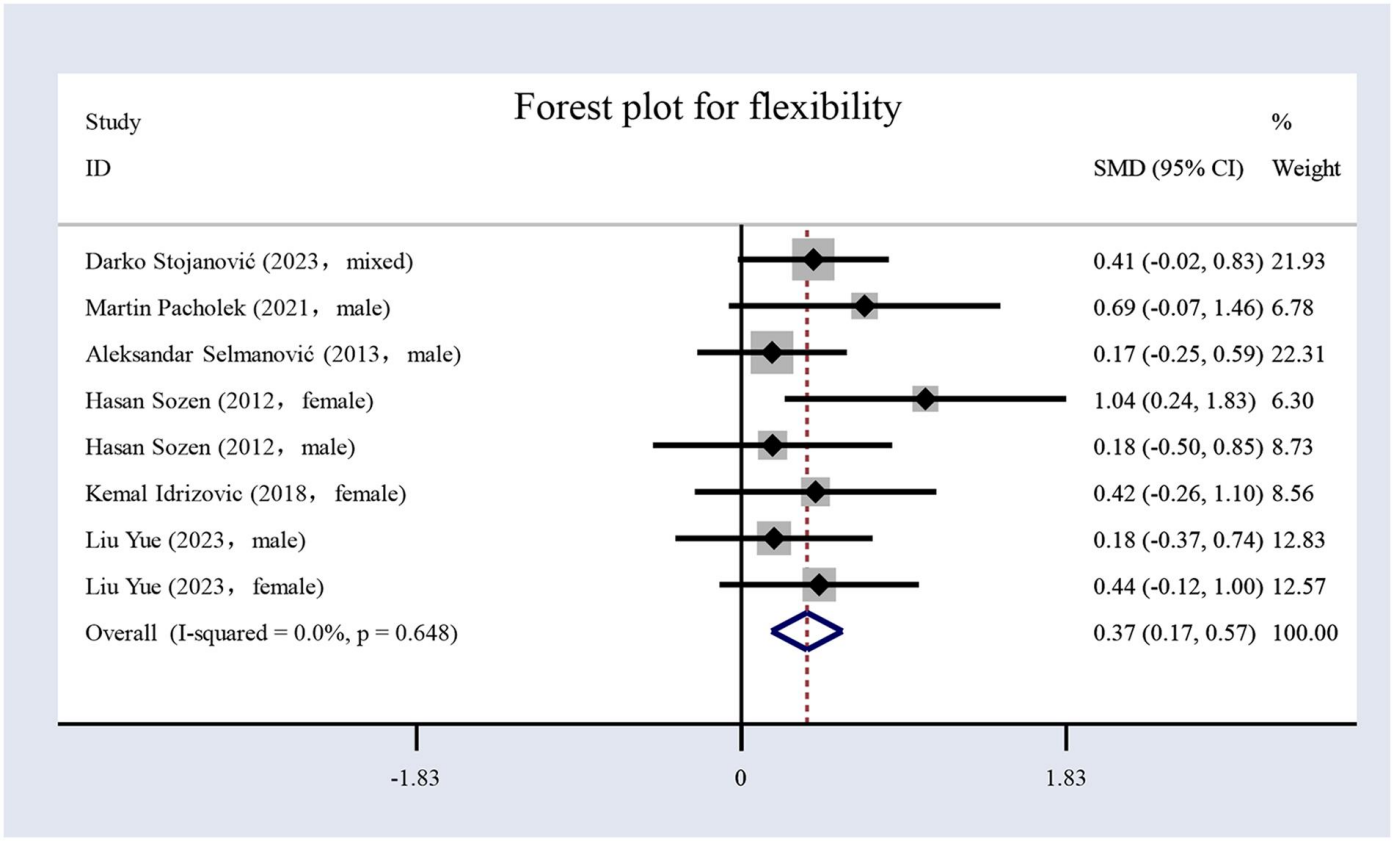
Forest plot for flexibility.

In the six included studies (17, 54, 57, 58, 60, 61), flexibility was assessed. The study comprised 400 participants overall (Table 1), including 196 in the EG and 204 in the CG. The forest plot for flexibility (Figure 7) illustrates that the results indicate that volleyball interventions significantly improved flexibility, with an *SMD* of 0.37 [95% (CI): 0.17 to 0.57, *p* < 0.001]. The heterogeneity was low (*I*² = 0%, *p* = 0.648).

#### 3.4.5 The impact of volleyball interventions on agility

**Figure 8:**
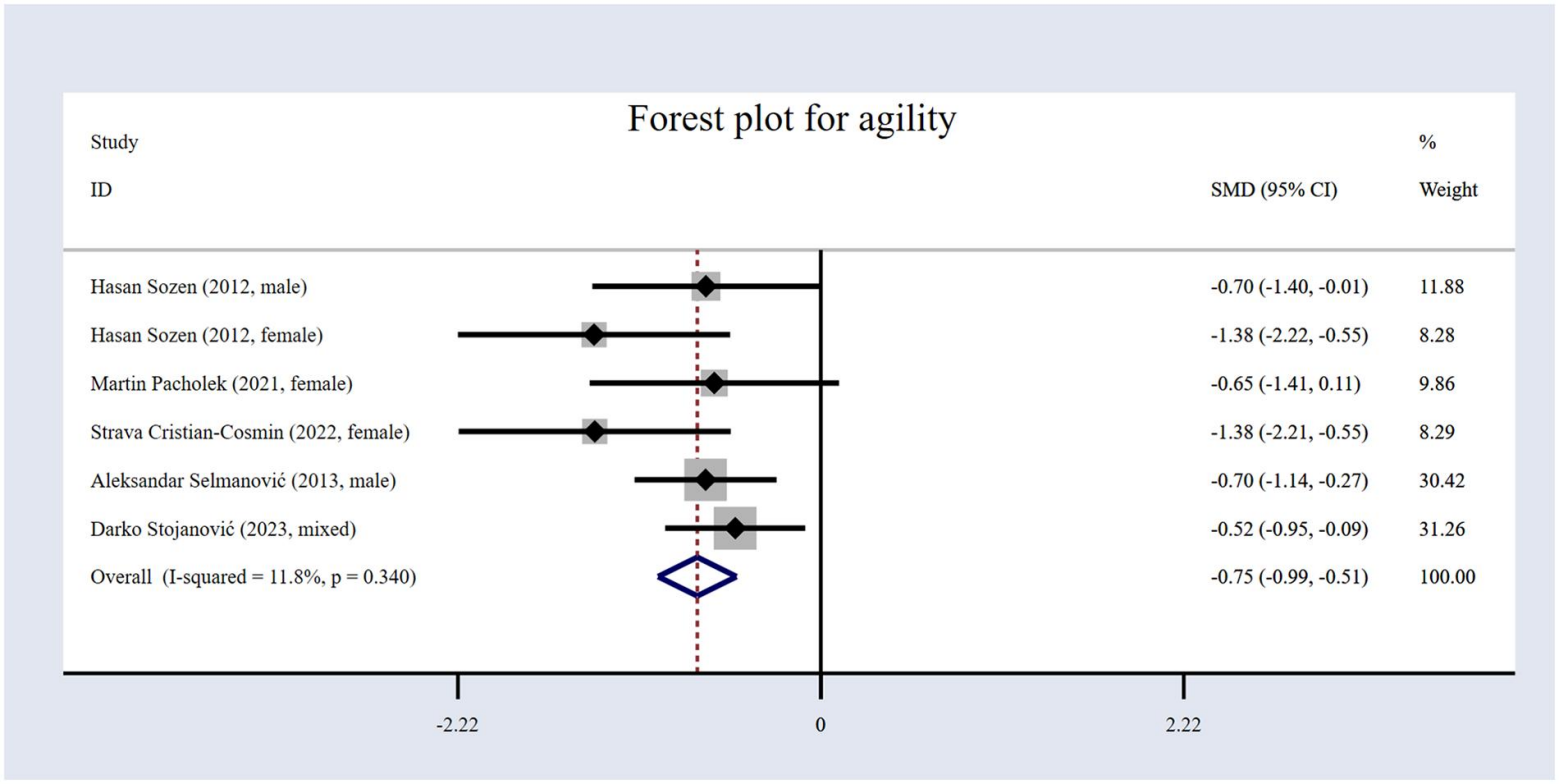
Forest plot for agility.

In the five included studies (17, 53, 54, 60, 61), agility was assessed. The study comprised 294 participants overall (Table 1), including 143 in the EG and 151 in the CG. The forest plot for agility (Figure 8) illustrates that the results indicate that volleyball interventions significantly improved agility, with an *SMD* of −0.75 [95% (CI): −0.99 to −0.51, *p* < 0.001]. The heterogeneity was low (*I*² = 11.8%, *p* = 0.34).

#### 3.4.6 The impact of volleyball interventions on speed

**Figure 9:**
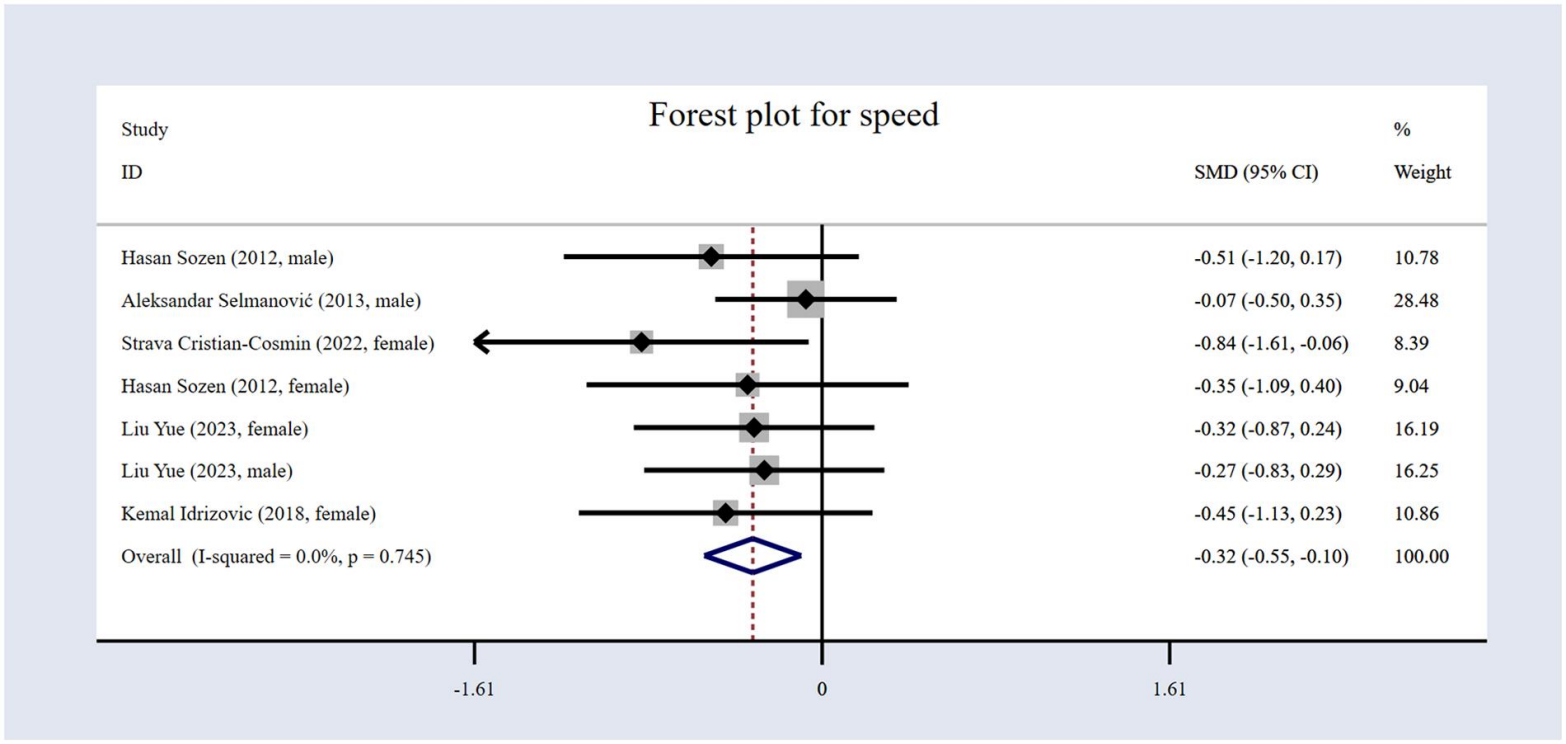
Forest plot for speed.

In the five included studies (17, 53, 57, 58, 60), speed was assessed. The study comprised 311 participants, as shown in Table 1: 157 in the EG and 154 in the CG. The forest plot for speed (Figure 9) illustrates that the results indicate that volleyball interventions significantly improved speed, with an *SMD* of −0.32 [95% (CI): −0.55 to −0.10, *p* = 0.005]. The heterogeneity was extremely low (*I*² = 0%, *p* = 0.745).

#### 3.4.7 The impact of volleyball interventions on body composition

**Figure 10:**
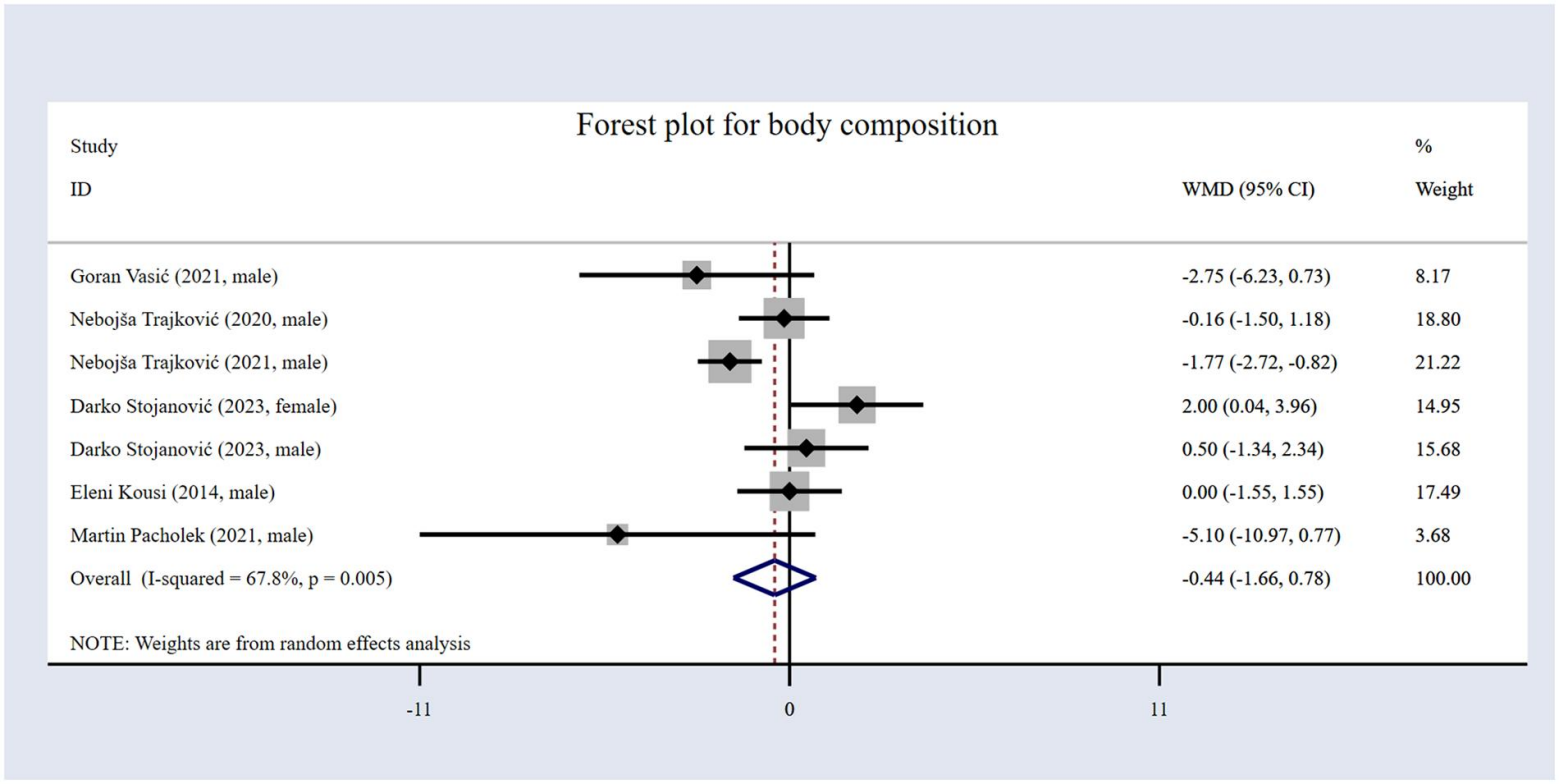
Forest plot for body composition.

The six included studies (16, 54–56, 59, 61) comprised 354 participants overall, as shown in Table 1, with 117 in the EG and 127 participants in the CG. The forest plot for body composition (Figure 10) illustrates that the results indicate that volleyball interventions did not significantly improve body composition, with a *WMD* of −0.44 [95% (CI): −1.66 to 0.78, *p* = 0.480]. Heterogeneity was high (*I*² = 67.8%; *p* = 0.005).

#### 3.4.8 The impact of volleyball interventions on power

**Figure 11:**
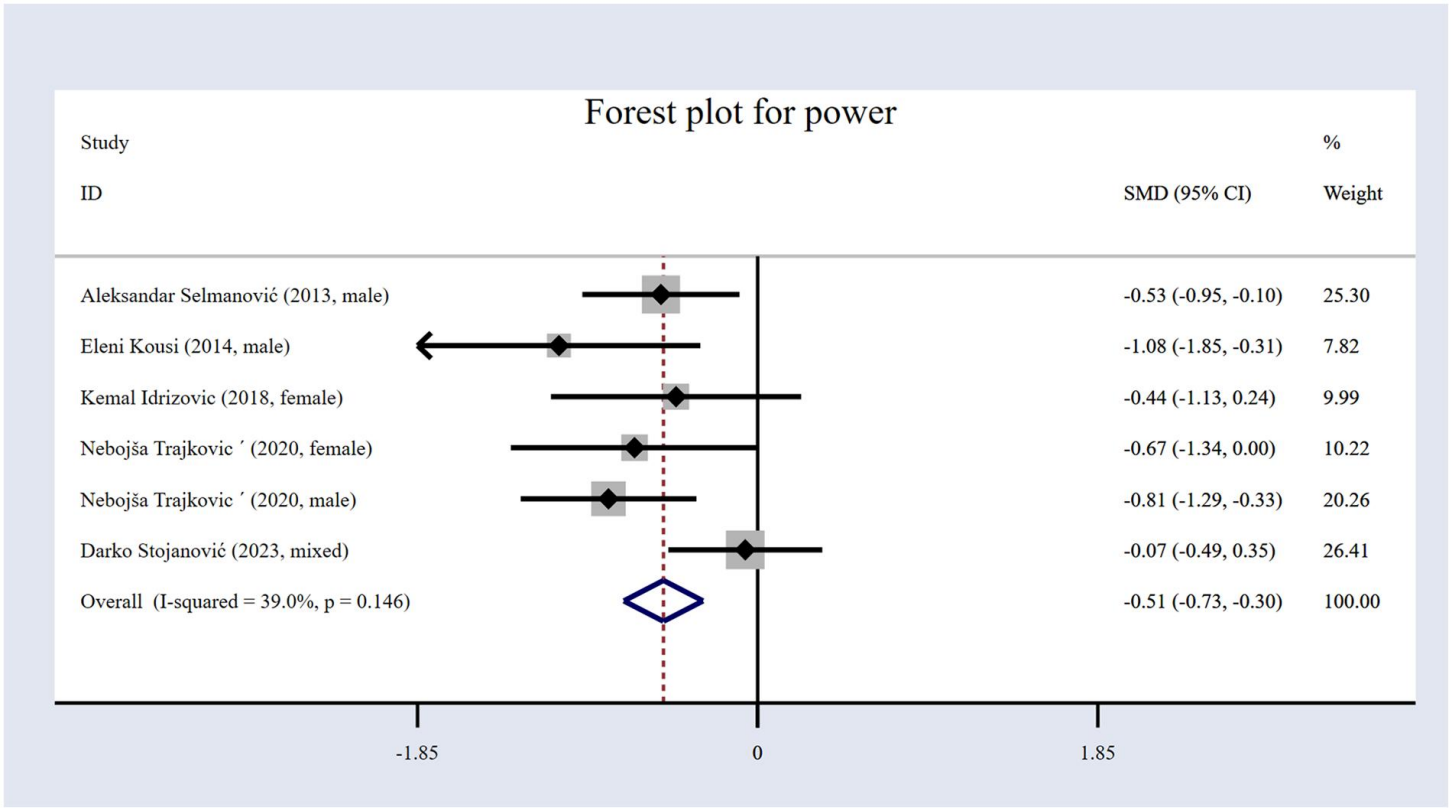
Forest plot for power.

The influence of volleyball interventions on power was examined through five studies (20, 58–61). Across these studies, 349 participants were involved (Table 1), with 171 assigned to the intervention group and 178 to the CG. The forest plot for power (Figure 11) reveals that volleyball interventions did produce a statistically meaningful effect on power, showing an *SMD* of −0.51 [95% (CI): −0.73 to −0.30, *p* < 0.001]. The results hint that volleyball training might benefit power, and the confidence interval remains below zero, suggesting statistical significance. Additionally, heterogeneity was moderate (I²= 39.0%, *p* = 0.146).

#### 3.4.9 The impact of volleyball interventions on balance

**Figure 12:**
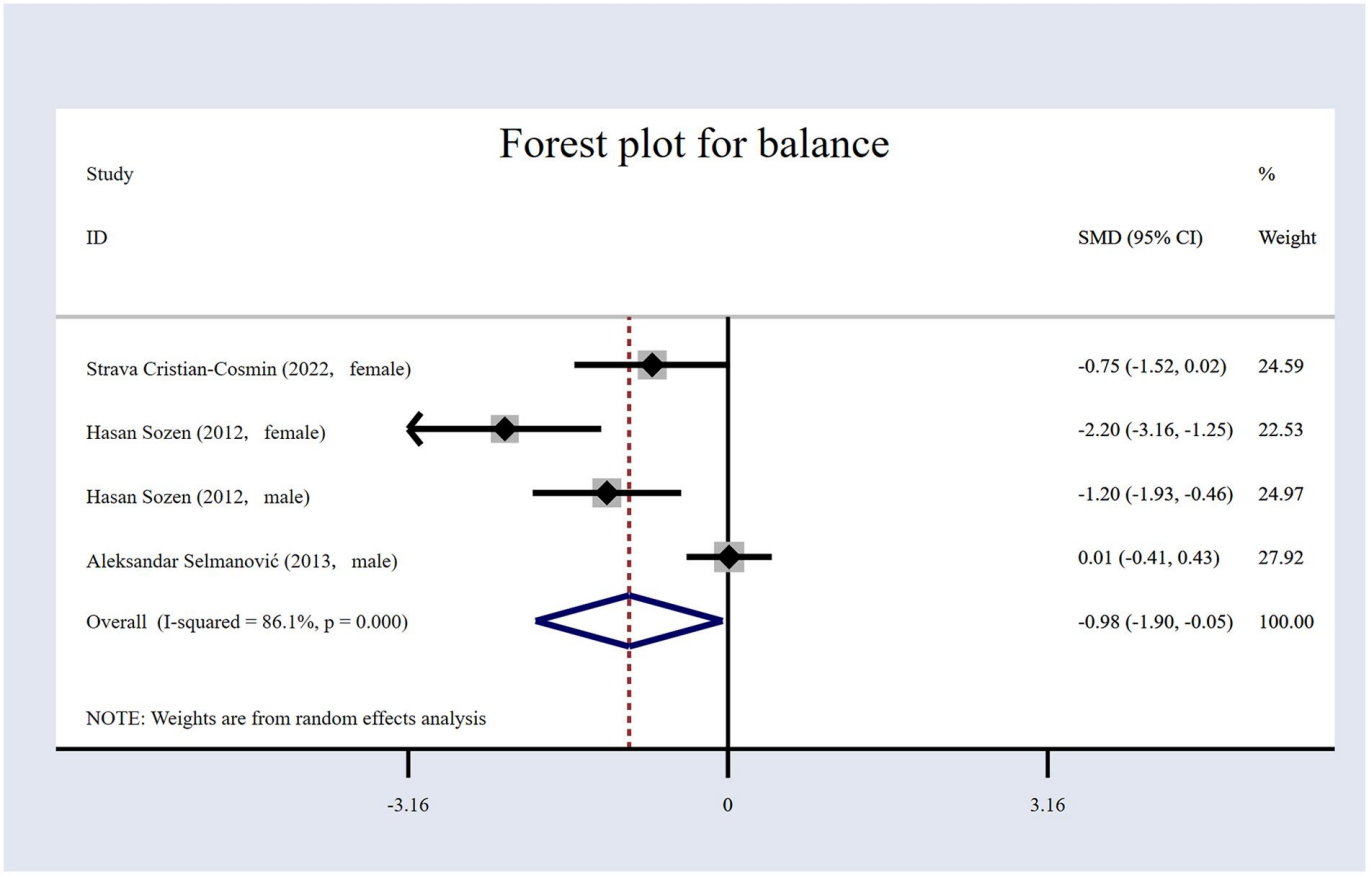
Forest plot for balance

The impact of volleyball interventions on balance was assessed in three studies. The study comprised 177 participants overall, as shown in Table 1, with 90 in the experimental group (EG) and 87 in the control group (CG). The forest plot for balance (Figure 12) indicates that volleyball interventions have a significant positive effect on balance, with an *SMD* of −0.98 [95% (CI): −1.90 to −0.05, *p* = 0.038]. This result suggests that the intervention group performed better in balance tests. This result suggests that the intervention group performed better in balance tests. The heterogeneity was high (I² = 86.1%, *p* = 0.001).

### 3.5 Publication bias analysis and sensitivity analysis

Publication bias was systematically evaluated using funnel plots and statistical tests (62–64) for all included studies. The funnel plots for outcomes such as muscular strength, flexibility, speed, body composition, balance, cardiovascular endurance, agility, and muscular endurance generally showed symmetrical distributions, suggesting a low risk of publication bias. Typically, the reliability of publication bias assessments improves when the number of included studies exceeds 10 (65). In the case of muscular strength, where over 10 studies were analysed, Begg's test (*p* = 0.112) and Egger's test (*p* = 0.471) both suggested that the results indicate an absence of significant publication bias.

These findings collectively suggest that the studies included in the meta-analysis provide a comprehensive and generally unbiased representation of the effects of volleyball interventions on various PF outcomes. The absence of significant publication bias in most outcomes enhances the credibility and reliability of the findings, supporting the conclusion that volleyball training positively impacts PF across multiple dimensions.

Subgroup analyses were not performed because the number of available studies for each outcome was limited. For outcomes with at least three included studies, leave-one-out sensitivity analyses were conducted to assess the robustness of the meta-analytic findings. Overall, these analyses did not materially alter the main conclusions of the meta-analysis, supporting the general robustness of the findings (see Supplementary Material S1: Sensitivity Analysis).

## 4 Discussion

### 4.1 Summary of findings

These twelve RCTs, involving a total of 656 participants, comprise this systematic review and meta-analysis, with 331 participants in the EG (203 males, 128 females) and 325 participants in the CG (202 males, 123 females). The intervention durations ranged from 4 weeks to 9 months, and training frequencies ranged from 1 to 4 times per week. The study evaluated the effects of the volleyball intervention on multiple physical fitness qualities. The results indicated that volleyball training had a significant positive effect on muscular endurance, cardiovascular endurance, muscular strength, flexibility, agility, speed, and balance, suggesting that it is efficacious in improving these PF qualities. However, the intervention did not show a significant impact on body composition.

Our research findings demonstrate that volleyball training significantly improves various aspects of PF, including muscular endurance, cardiorespiratory endurance, muscular strength, and power. For muscular endurance, the included studies most used sit-ups as a representative test. Hasan Sozen (17) found that students who received volleyball training achieved significantly better sit-up performance than those who did not participate in volleyball training. Similar improvements were also reported in overweight and obese girls after a 6-month volleyball programme (53). Liu et al. (57) further showed that, after one semester of volleyball training, female university students significantly improved their one-minute sit-up performance. These findings suggest that volleyball training can effectively enhance trunk and core muscular endurance, likely because the sport requires repeated defensive postures, ready positions, rapid transitions, and sustained activation of the trunk and hip musculature (17, 53, 57).

Volleyball training also showed beneficial effects on cardiorespiratory endurance. Across the included studies, this outcome was assessed using representative measures, including the Yo-Yo Intermittent Recovery Test, VO₂max, and running performance. Trajković et al. (20) reported that after an 8-month volleyball intervention, adolescents improved their YYIRT1 performance, while Stojanović et al. (61) found significant improvements in VO₂max after a 16-week school-based TGfU volleyball intervention. Liu et al. (57) also reported that, after one semester of volleyball training, the 50 m, 800 m, and 1000 m running performance of university students improved significantly. These results indicate that volleyball training can enhance cardiorespiratory endurance, likely because it is an intermittent sport involving repeated bouts of moderate-to-high-intensity movement interspersed with short recovery periods (20, 57, 61).

Concerning muscular strength and power, our findings also support a positive training effect of volleyball. For example, Trajković et al. (20) reported that after 8 months of volleyball, vertical jump performance improved by 3.0% (ES = 0.17), which was attributed to the numerous jumps athletes perform during a volleyball game. Similarly, Trajković et al. (20) found that boys and girls in the experimental group improved their medicine ball throw performance by 4.9% (ES = 0.42) and 6.1% (ES = 0.30), respectively.

In a study by Stojanović et al. (61), the volleyball group showed increases of 9.1% and 9.9% in squat jump and countermovement jump performance, respectively. Liu et al. (57) also reported improvements in standing long jump and pull-up performance after one semester of volleyball training. These findings demonstrate that volleyball training improves both muscular strength and explosive power, which are essential for executing high-intensity actions such as jumping, spiking, blocking, and overhead hitting.

These improvements in muscular endurance, cardiorespiratory endurance, muscular strength, and power from volleyball training are not only crucial for athletic performance but also have practical implications for the daily lives and overall health of the general population (66). Enhancements in muscular endurance and strength increase stamina, helping individuals perform repetitive movements more easily in everyday activities, such as lifting heavy objects, climbing stairs, and doing household chores. Improved cardiorespiratory endurance increases tolerance for daily physical activities, reduces fatigue, and enhances overall quality of life (67).

Increased explosive power improves athletic performance and reaction time, and helps prevent accidental falls and facilitates quick bodily adjustments (68). Overall, volleyball training is not only a means of improving athletic performance but also a beneficial form of exercise for the general population that can enhance physical fitness and help prevent common injuries in everyday life.

In addition to improvements in muscular endurance, cardiorespiratory endurance, muscular strength, and power, our research findings demonstrate that volleyball training also significantly affects flexibility, agility, speed, and balance. Research found that after training, university students significantly improved their sit-and-reach scores, with male students increasing from 9.994 ± 7.215 cm to 14.792 ± 7.255 cm and female students from 10.565 ± 2.480 cm to 18.845 ± 5.804 cm (57). This improvement in flexibility is crucial in volleyball, as it enhances the ability to perform dynamic movements and reduces the risk of injury.

Taware, Bhutkar, and Surdi noted that volleyball requires athletes to move in multiple directions to reach the ball, thereby requiring good flexibility and muscular endurance (69).

For speed, the included studies used representative measures such as the 10 × 5 m shuttle-run, 20 m sprint, and 50 m run. Hasan Sozen (17) found that female students who received volleyball training performed significantly better in the 10 × 5 m shuttle-run than sedentary controls, and a significant difference was also observed in the total sample.

Similarly, Strava Cristian-Cosmin et al. (53) reported that overweight and obese girls significantly improved their 10 × 5 m shuttle-run performance after a 6-month volleyball programme. Liu et al. (57) further showed that university students significantly reduced their 50 m sprint time after one semester of volleyball training. These findings suggest that volleyball training can enhance short-distance speed, probably because players must repeatedly perform short accelerations, rapid court coverage, and quick reactive movements during play.

Agility was also improved following volleyball training. This multidirectional movement is fundamental in volleyball, requiring quick changes of direction, jumps, and dives to improve agility and overall physical coordination. Stojanović et al. (61) demonstrated that students in the volleyball group improved agility by 1.8%, as assessed by the agility T-test. Selmanovi and Milanovi (60) also included a 20-yard agility test and described defensive movements, lateral movements, tactical drills, and mini-volleyball games as key elements of the intervention. In addition, Strava Cristian-Cosmin et al. (53) found significant improvement in the shuttle-run test after volleyball training. Speed and agility are crucial for volleyball, enabling players to react quickly to the ball, execute effective serves and spikes, and cover the court efficiently.

Hasan Sozen (17) found that volleyball players had significantly better balance than a control group who did not participate in volleyball training, as assessed by the Flamingo Balance Test, highlighting the critical role of balance in volleyball for maintaining stability during jumps, landings, and rapid directional changes, thus reducing the risk of falls and related injuries. These PF enhancements are directly attributable to the high-intensity, intermittent nature of volleyball training, which involves short bursts of rapid movement followed by periods of lower intensity. These results align with our findings, confirming that volleyball training effectively enhances flexibility, agility, speed, and balance. These improvements in flexibility, agility, speed, and balance have equally significant health-promoting and quality-of-life implications for the general population.

Good flexibility helps reduce the risk of muscle strains and joint injuries in everyday life (70). Increased agility and speed enhance the efficiency of daily activities and the ability to cope with unexpected situations, such as making quick adjustments in movement and preventing falls (71). Improved balance enhances stability, particularly in activities such as walking and climbing stairs, thereby reducing the risk of accidental injuries, including falls (72). Overall, improvements in these physical fitness qualities not only optimise athletic performance but also have a positive impact and offer practical guidance for daily life and long-term health management in the general population.

However, although volleyball training significantly improved several aspects of PF, its effect on BMI was not statistically significant in the present study. Possible explanations include insufficient intervention duration, inadequate training intensity, or relatively low baseline body weight and BMI among participants. High heterogeneity was observed for muscular endurance (I²= 84.9%), balance (I²= 86.1%), and body composition (I²= 67.8%). For body composition, the observed heterogeneity may be related to differences in baseline weight status, age, sex composition, and intervention duration across studies. For muscular endurance, heterogeneity may be attributable to differences in participant characteristics and intervention exposure, as the included studies involved different age groups and intervention periods ranging from 4 weeks to one semester or 9 months (54, 60).

Nevertheless, the leave-one-out sensitivity analysis indicated that the pooled estimate for muscular endurance remained relatively stable, suggesting that the overall finding was generally robust. For balance, the heterogeneity appeared to be more substantial and may be explained by marked differences in participant characteristics, intervention exposure, and assessment methods across studies. Specifically, Hasan Sozen (17) included older secondary school students of both sexes who participated in the school volleyball team in addition to regular physical education classes and assessed balance using the Flamingo Balance Test, whereas Selmanovi & Milanovi (60) included only 11-year-old boys who received one additional 45-minute volleyball session per week for 9 months and assessed balance using the low beam stand test. These clinical and methodological differences, together with the greater improvement reported in Hasan Sozen (17), may have contributed to the substantial heterogeneity in the balance outcome.

Furthermore, gender-specific physiological responses to physical fitness interventions may have contributed to this heterogeneity, as males and females can exhibit different patterns of adaptation. These sources of variability highlight the complexity of achieving consistent outcomes across studies and suggest that standardising participant criteria, intervention protocols, and assessment measures could improve the comparability of results in future research.

### 4.2 Limitations

Although the present study provides evidence supporting the effects of volleyball interventions on physical fitness, several limitations should be acknowledged. First, the number of studies included in this review was relatively small (*n* = 12), with an overall sample size of 656 participants. In addition, some specific outcomes were based on only a few studies, such as balance and muscular endurance. This may have reduced the statistical power and stability of the pooled estimates, making these findings more susceptible to the influence of individual studies. The limited sample size may also restrict the generalisability of the findings. Second, the training protocols used in the included studies varied, and the content and intensity of the interventions may have differed considerably across studies, contributing to inconsistencies in the findings. Third, differences in participant characteristics, such as age, sex, and population group (e.g., adolescents, students, and adults), may affect the applicability of the findings. Fourth, intervention duration varied substantially across studies, ranging from 4 weeks to 9 months. This variation may have influenced the observed effects on PF, indicating the need for further well-designed studies to determine the optimal duration of volleyball interventions.

### 4.3 Research advantage

Several notable strengths characterise this systematic review and meta-analysis. First, including a diverse range of RCTs across different populations enhances the clinical significance and applicability of the results. Second, a comprehensive search strategy across these databases ensured the inclusion of all relevant studies in this paper. Thirdly, the strength and reliability of the findings were enhanced through rigorous literature searches, screening, and bias assessment, ensuring the accuracy and scientific validity of the studies. In addition, detailed data extraction methods and consistent results across studies further validated the conclusions drawn from the meta-analysis. Ultimately, integrating qualitative and quantitative analyses facilitated a comprehensive understanding of the impact of volleyball interventions on physical fitness (PF). Furthermore, the results of this study offer practical insights for educators, coaches, and health practitioners seeking to design volleyball-based programmes that improve physical fitness. The identified improvements in cardiovascular endurance, muscular strength, balance, and flexibility across diverse populations underscore volleyball's potential as a versatile and effective health-promotion intervention.

## 5 Conclusion

This meta-analysis suggests that volleyball interventions are an effective form of training that significantly enhances various physical attributes, including flexibility, balance, muscular endurance, muscular strength, agility, and cardiovascular endurance. These positive effects have been confirmed across different populations, including primary school students, adolescents, college and university students, and untrained adults. Based on these findings, we propose volleyball intervention protocols to guide practical applications and inform future research. The available evidence suggests that effective volleyball-based training programmes may last 4 to 36 weeks, with a training frequency of 1 to 4 sessions per week and each session lasting 40 to 90 min. Intervention methods include volleyball-specific training, TGFU volleyball training, VSBT, and MRV. While these parameters provide a helpful starting point, further research is warranted to determine optimal combinations of training frequency, duration, and intensity for specific populations, including adolescents, university students, and adults of different sexes and varying fitness levels. Future research should investigate the optimal intervention period, frequency, duration, and intensity for specific age groups and identify the most effective volleyball-based interventions. Additionally, attention should be paid to the interactions among these training variables to further enhance PF, particularly in specific populations.

## Data Availability

The original contributions presented in the study are included in the article/Supplementary Material, further inquiries can be directed to the corresponding author.
