# Supplementary figures and images for: Evaluating volleyball interventions for enhancing physical fitness in healthy individuals: a systematic review and meta-analysis
Source: Front Sports Act Living. 2026 May 12;8:1800110. doi: 10.3389/fspor.2026.1800110 (PMC13201486; doi:10.3389/fspor.2026.1800110)

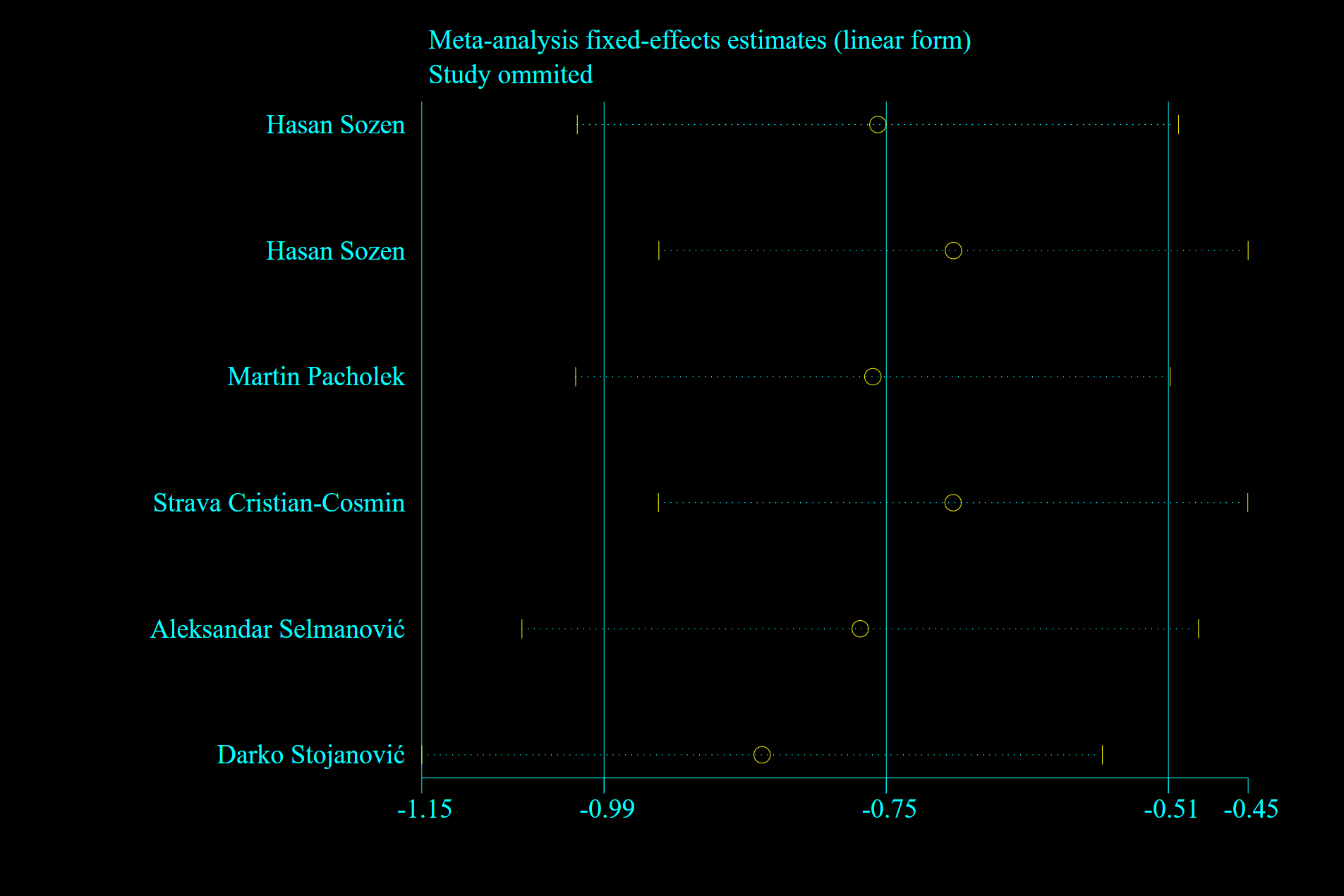

Supplement: Supplementary file 1 [file Datasheet1.zip › Supplementary files/S1 Leave-one-out sensitivity analysis/Agility-Leave-one-out sensitivity analysis.tif]

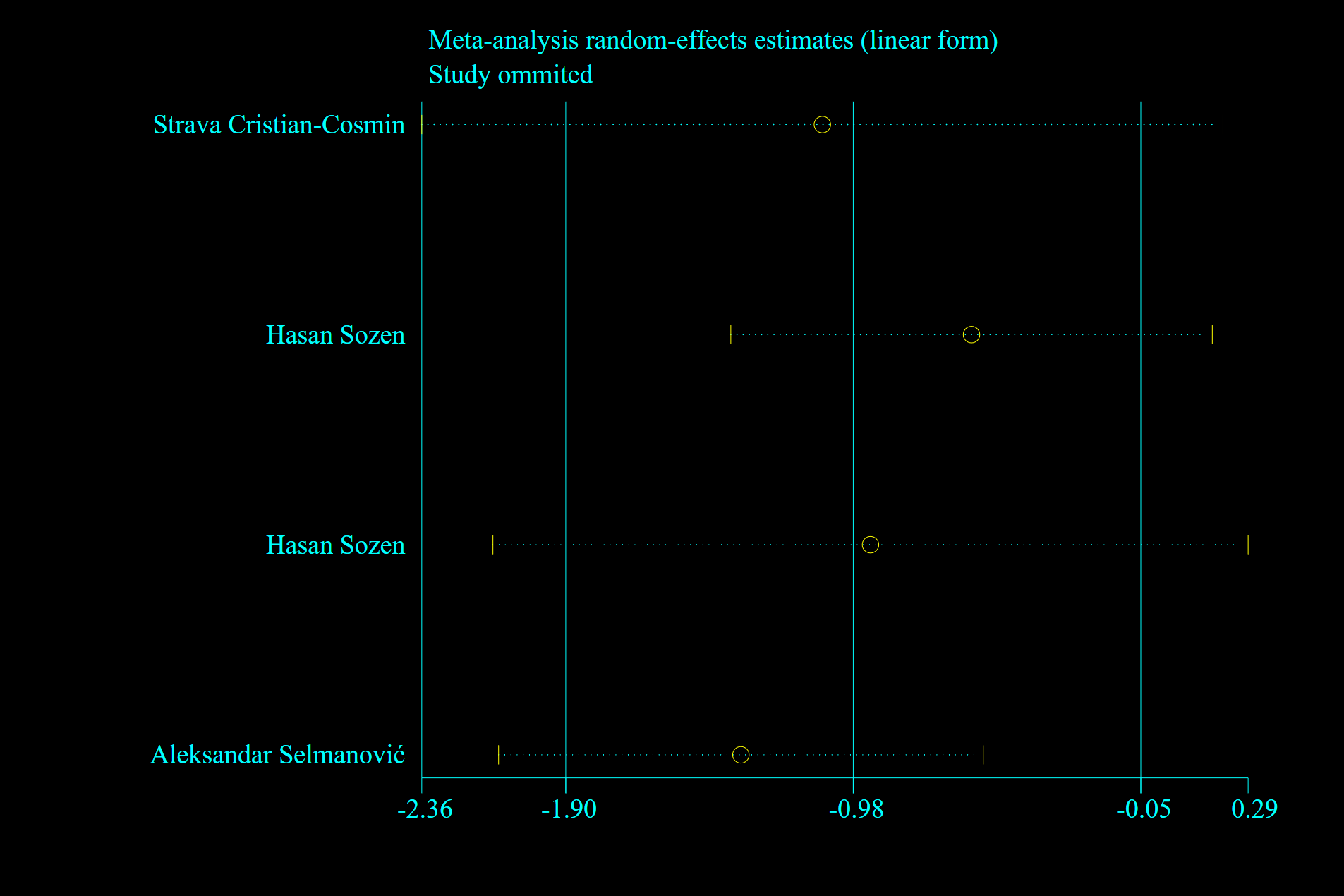

Supplement: Supplementary file 1 [file Datasheet1.zip › Supplementary files/S1 Leave-one-out sensitivity analysis/Balance-Leave-one-out sensitivity analysis.tif]

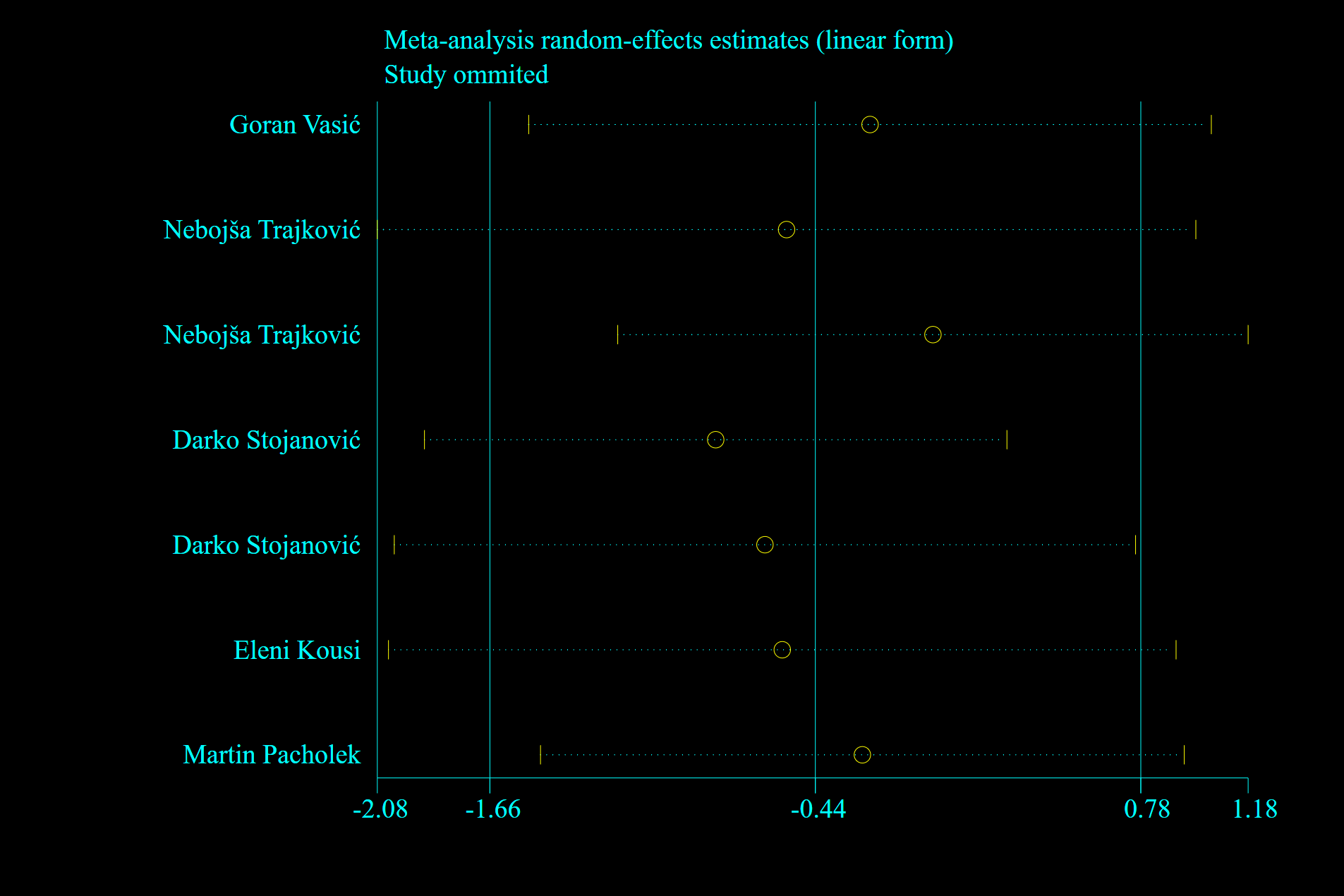

Supplement: Supplementary file 1 [file Datasheet1.zip › Supplementary files/S1 Leave-one-out sensitivity analysis/Body Composition-Leave-one-out sensitivity analysis.tif]

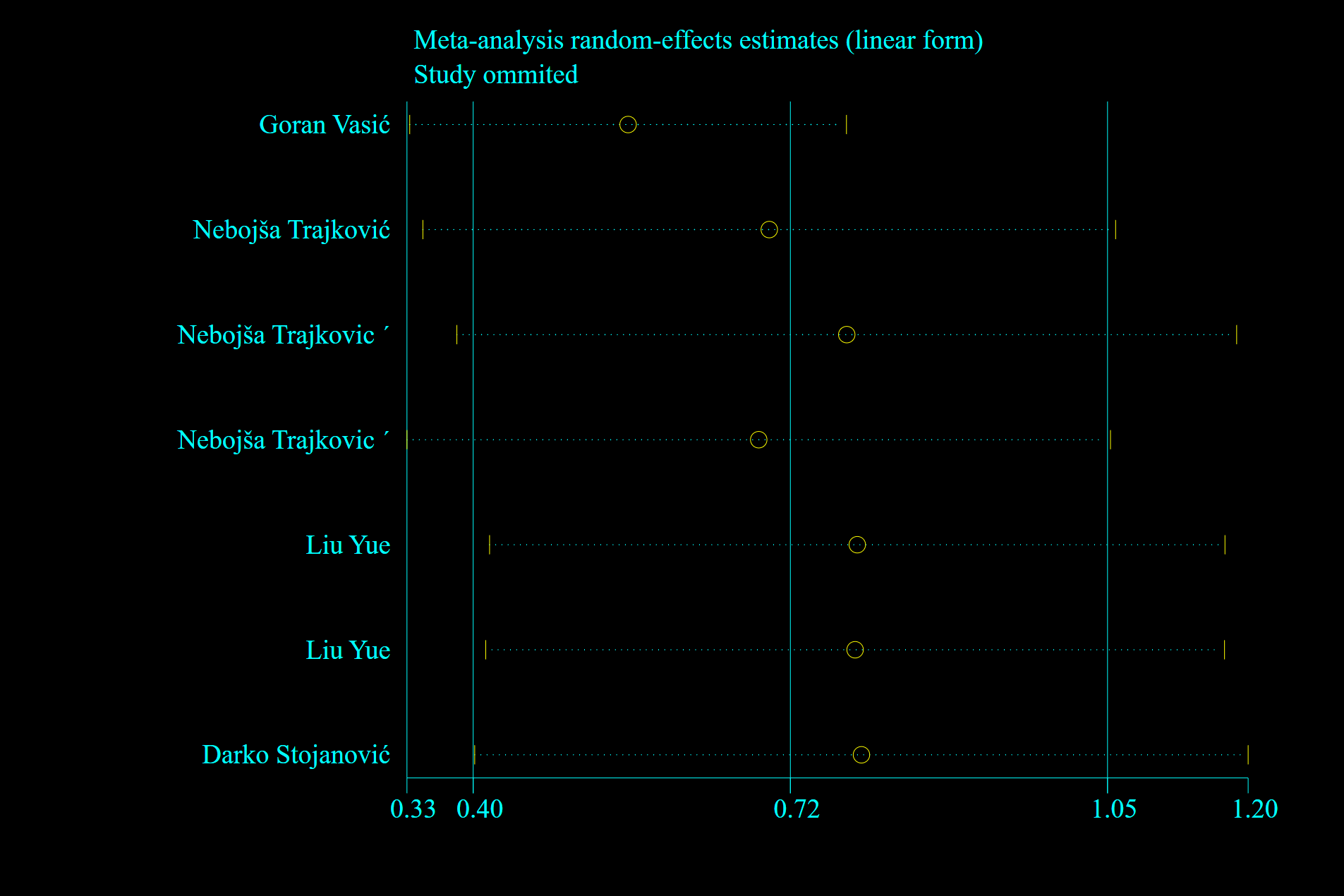

Supplement: Supplementary file 1 [file Datasheet1.zip › Supplementary files/S1 Leave-one-out sensitivity analysis/Cardiovascular Endurance-Leave-one-out sensitivity analysis.tif]

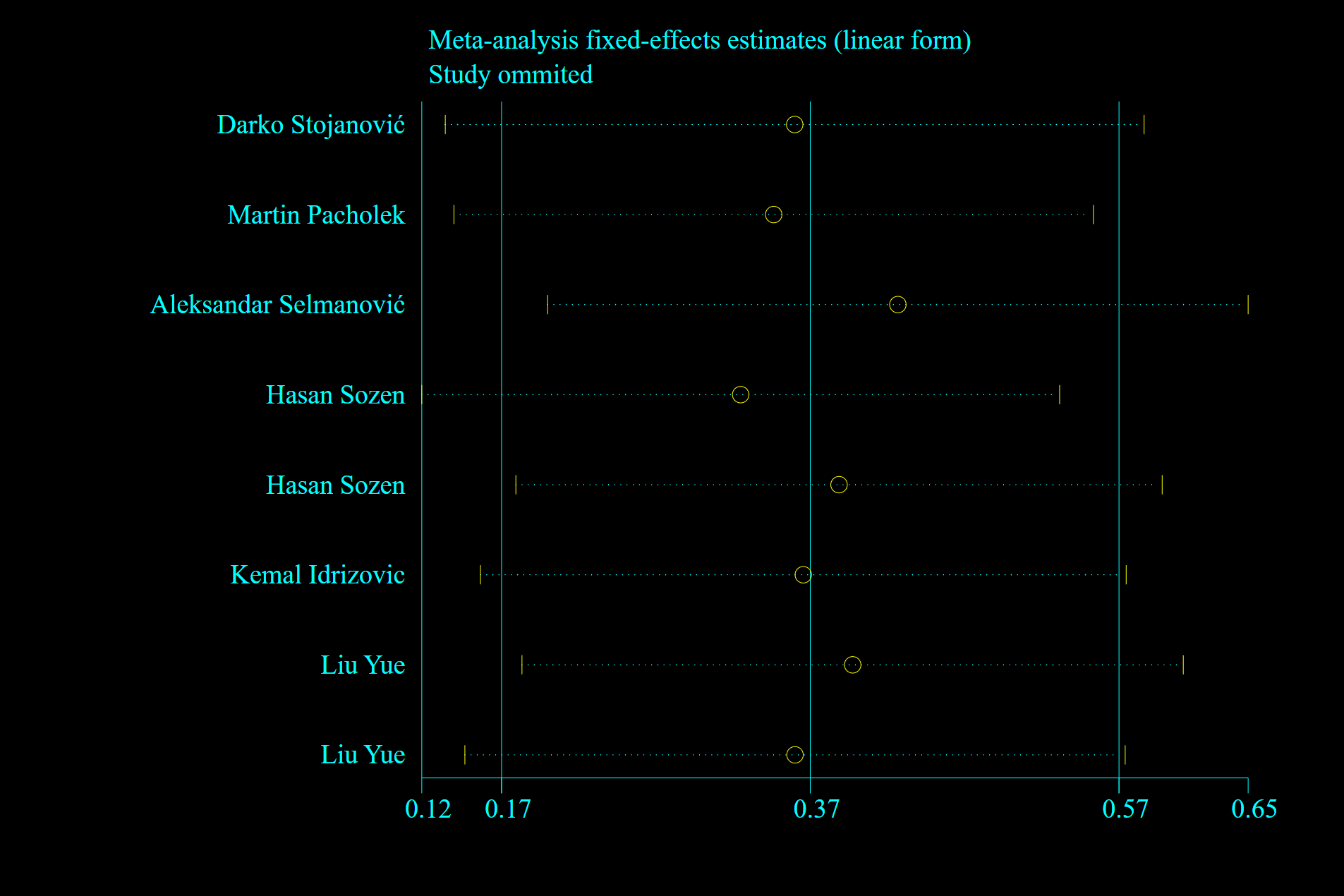

Supplement: Supplementary file 1 [file Datasheet1.zip › Supplementary files/S1 Leave-one-out sensitivity analysis/Flexibility-Leave-one-out sensitivity analysis.tif]

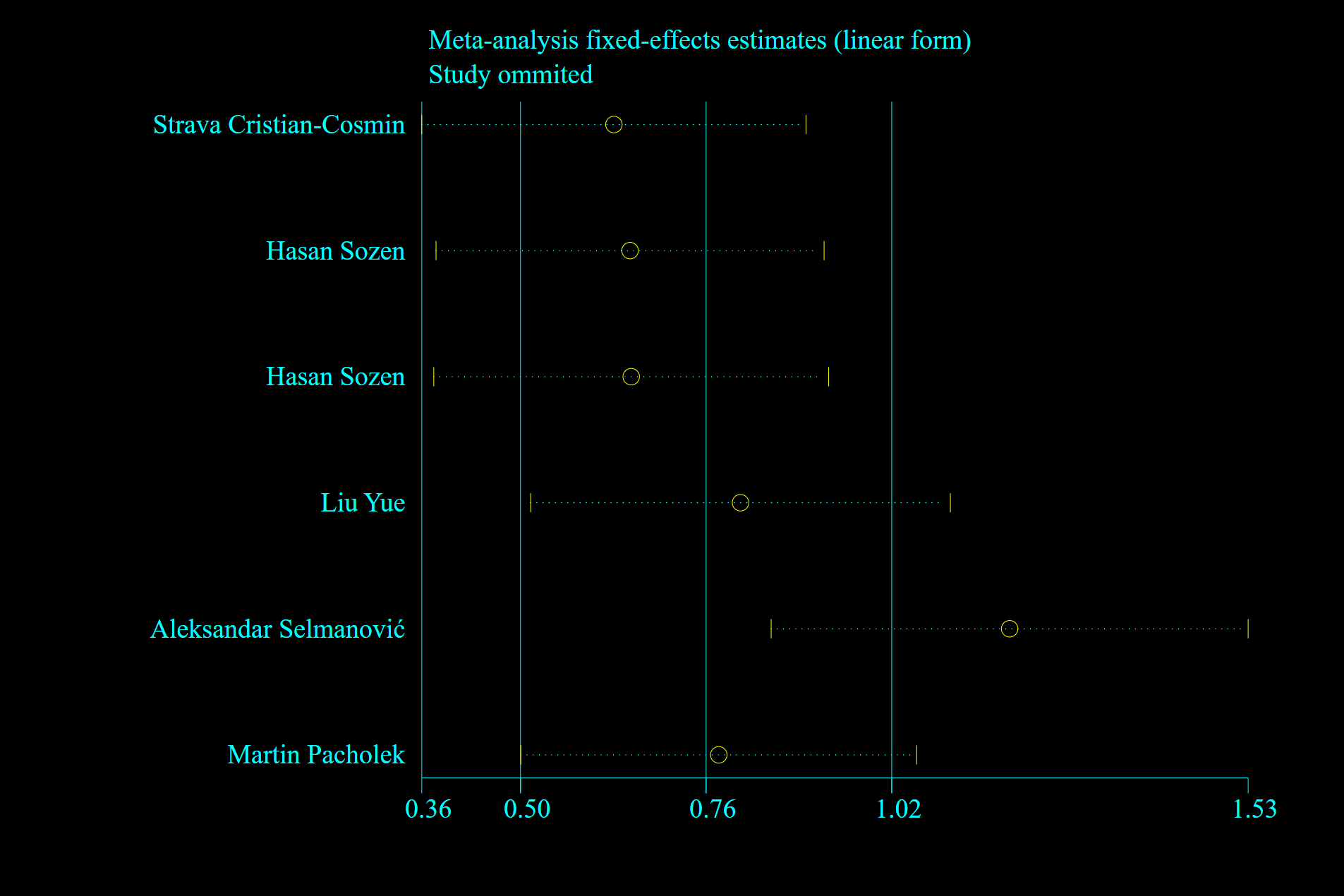

Supplement: Supplementary file 1 [file Datasheet1.zip › Supplementary files/S1 Leave-one-out sensitivity analysis/Muscular Endurance-Leave-one-out sensitivity analysis.tif]

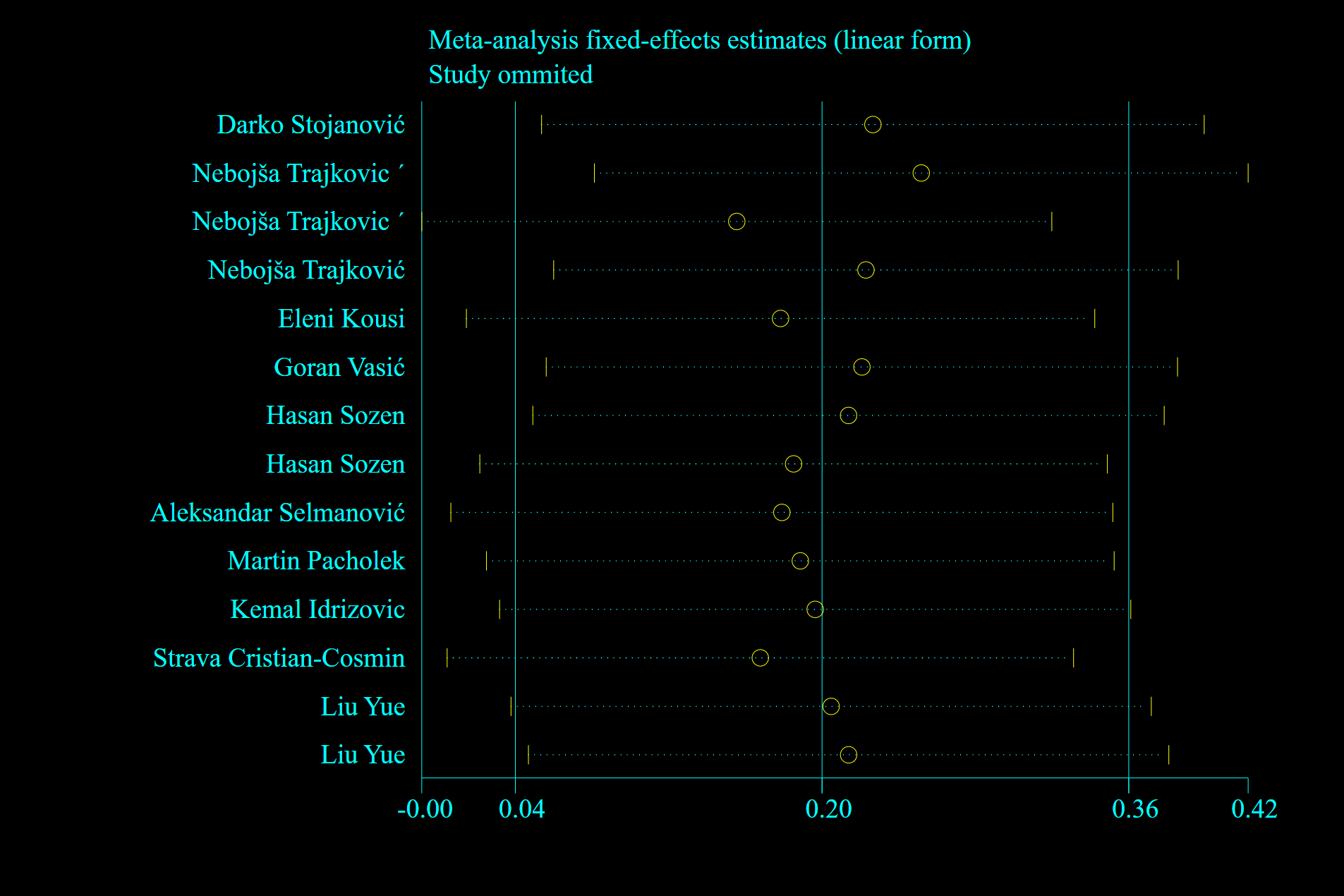

Supplement: Supplementary file 1 [file Datasheet1.zip › Supplementary files/S1 Leave-one-out sensitivity analysis/Muscular Strength--Leave-one-out sensitivity analysis.tif]

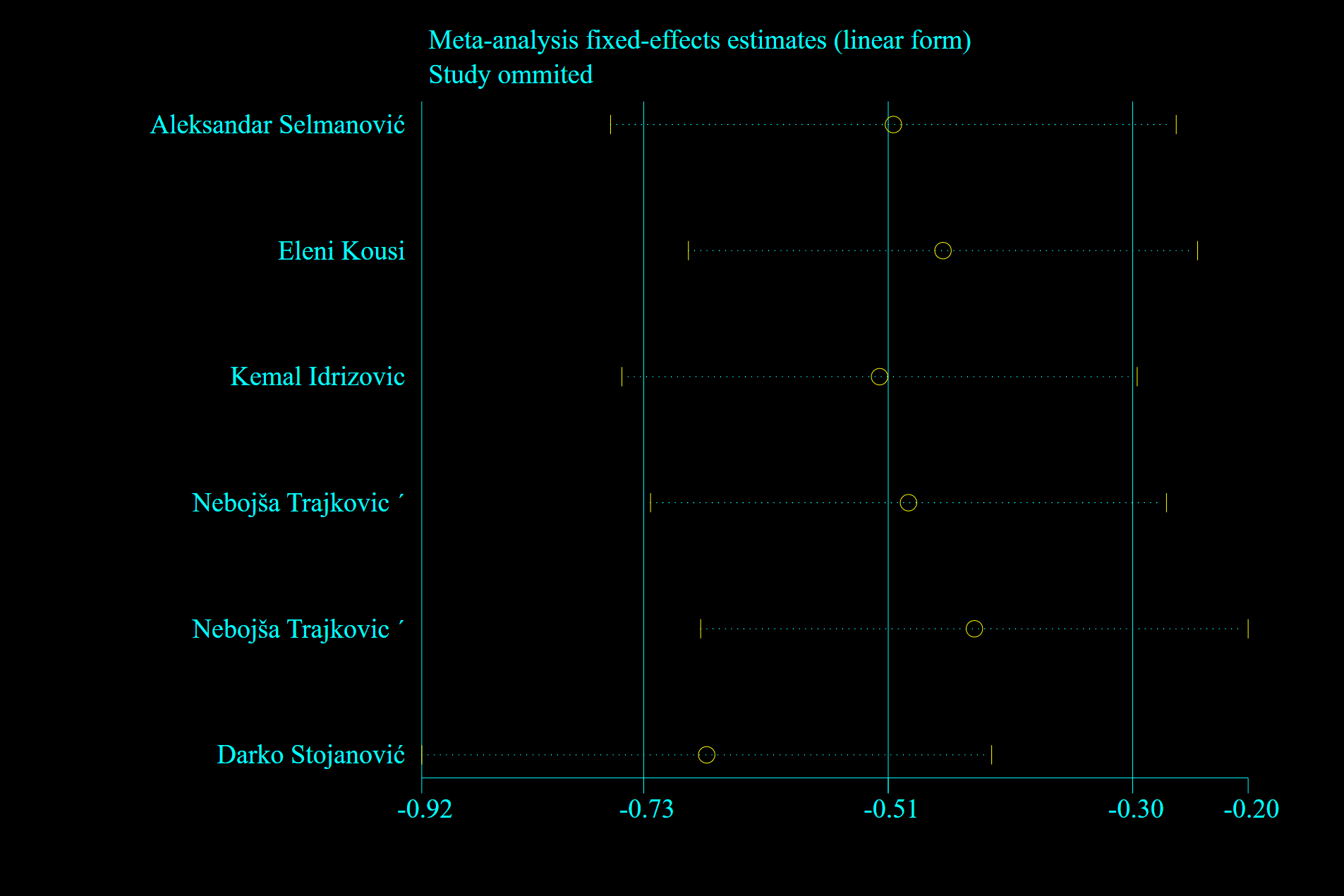

Supplement: Supplementary file 1 [file Datasheet1.zip › Supplementary files/S1 Leave-one-out sensitivity analysis/Power-Leave-one-out sensitivity analysis.tif]

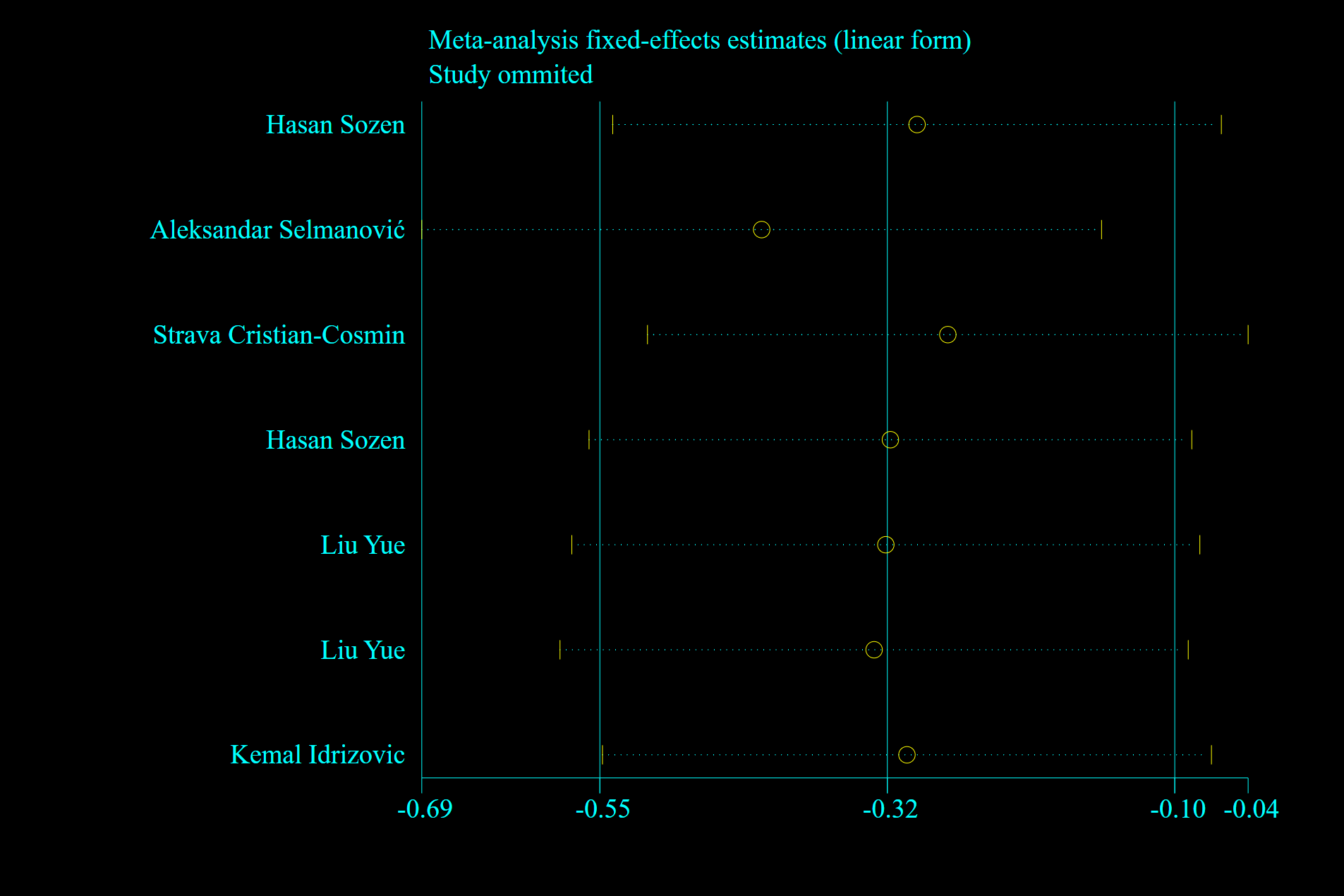

Supplement: Supplementary file 1 [file Datasheet1.zip › Supplementary files/S1 Leave-one-out sensitivity analysis/Speed-Leave-one-out sensitivity analysis.tif]

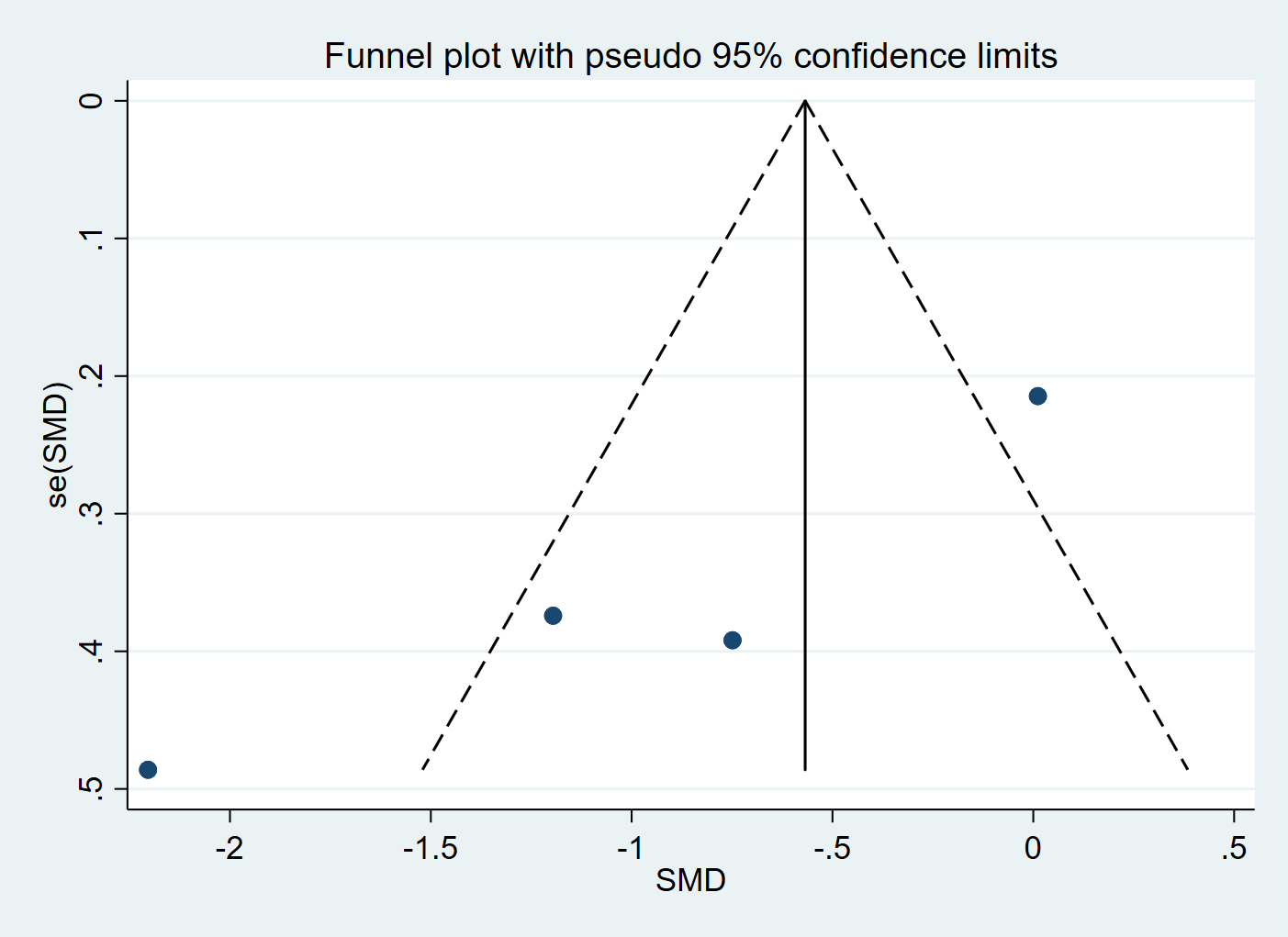

Supplement: Supplementary file 1 [file Datasheet1.zip › Supplementary files/S2 Funnel plot/Funnel plot Balance.tif]

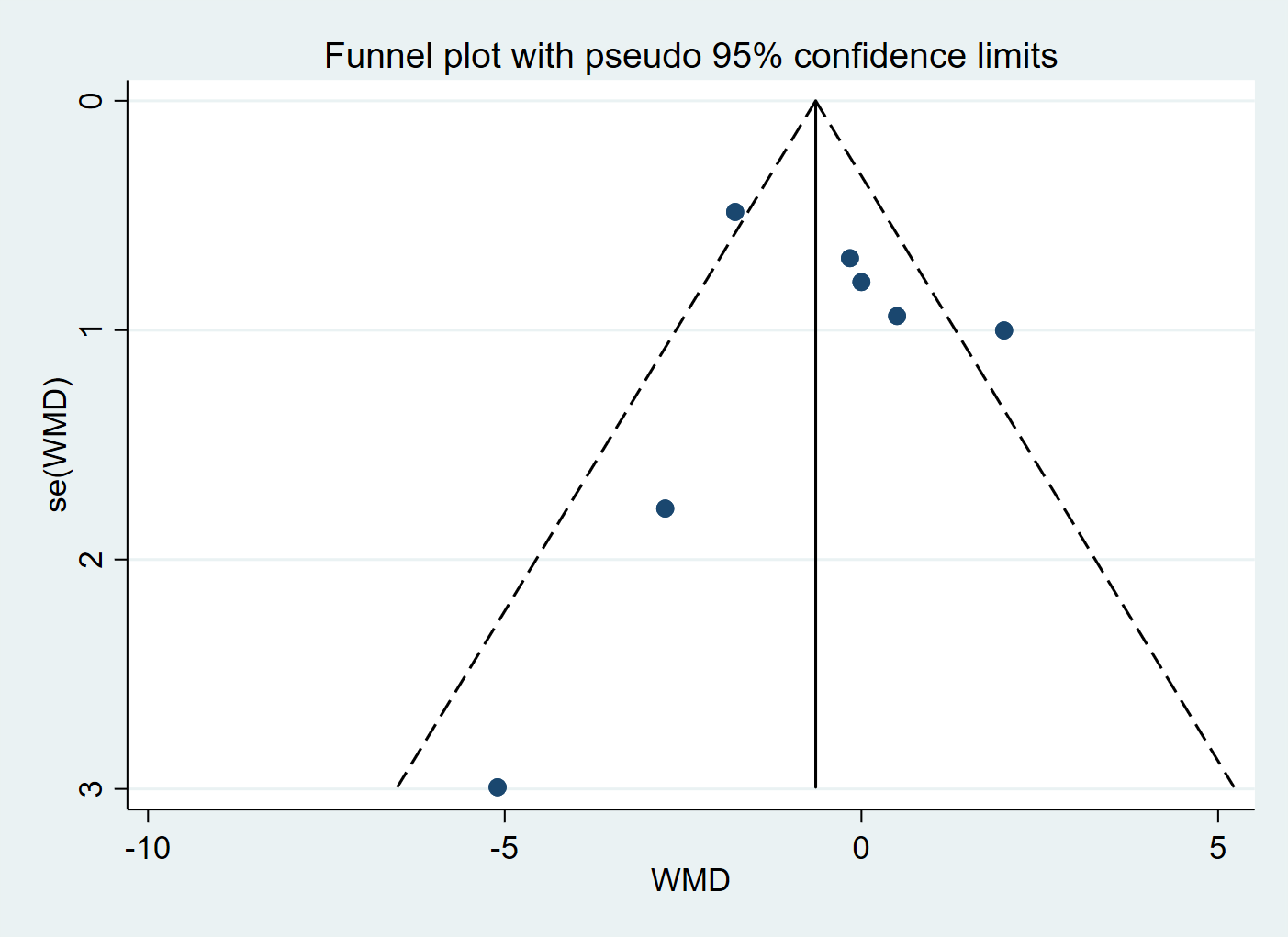

Supplement: Supplementary file 1 [file Datasheet1.zip › Supplementary files/S2 Funnel plot/Funnel plot Body Composition.tif]

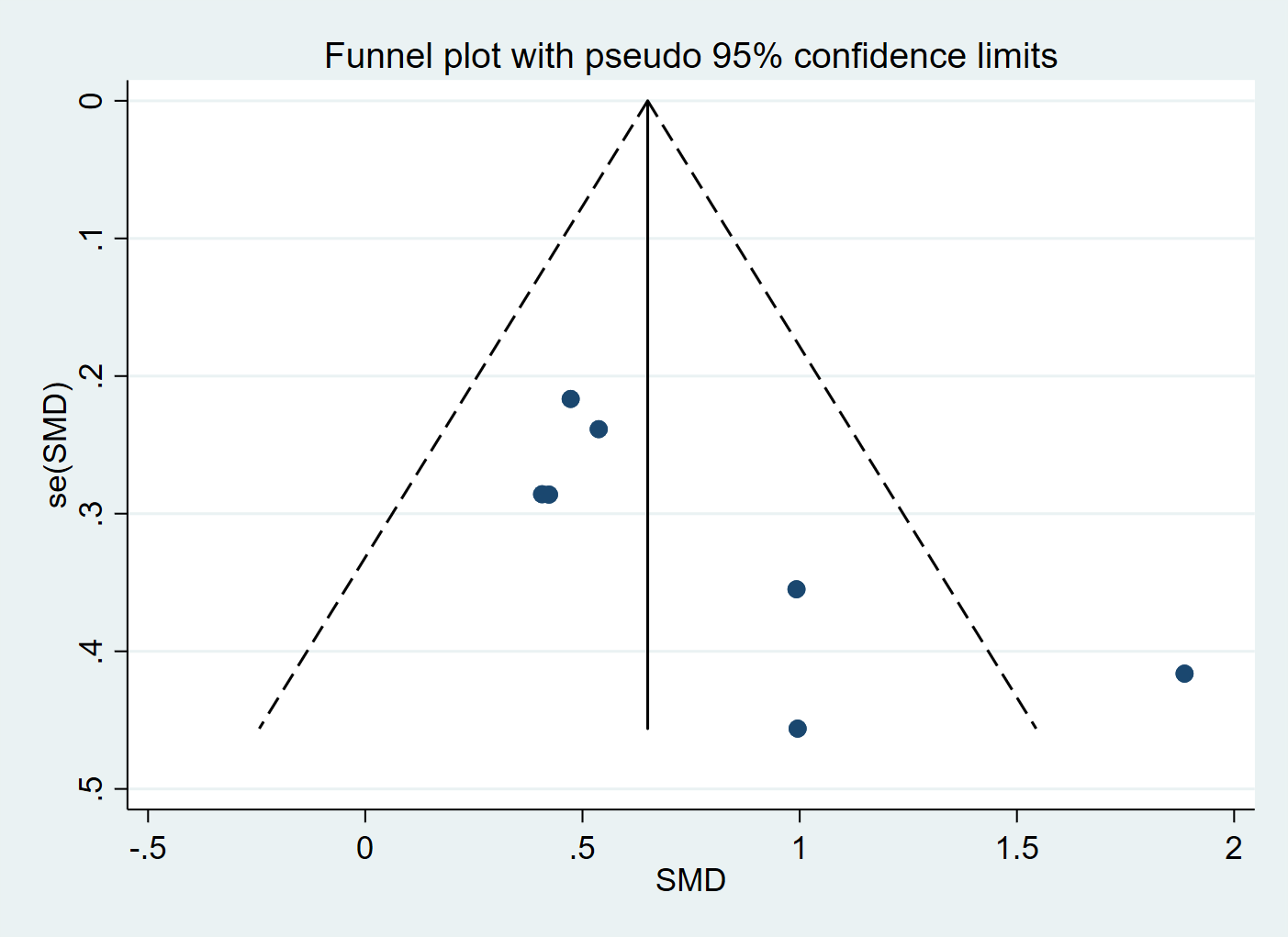

Supplement: Supplementary file 1 [file Datasheet1.zip › Supplementary files/S2 Funnel plot/Funnel plot Cardiovascular Endurance.tif]

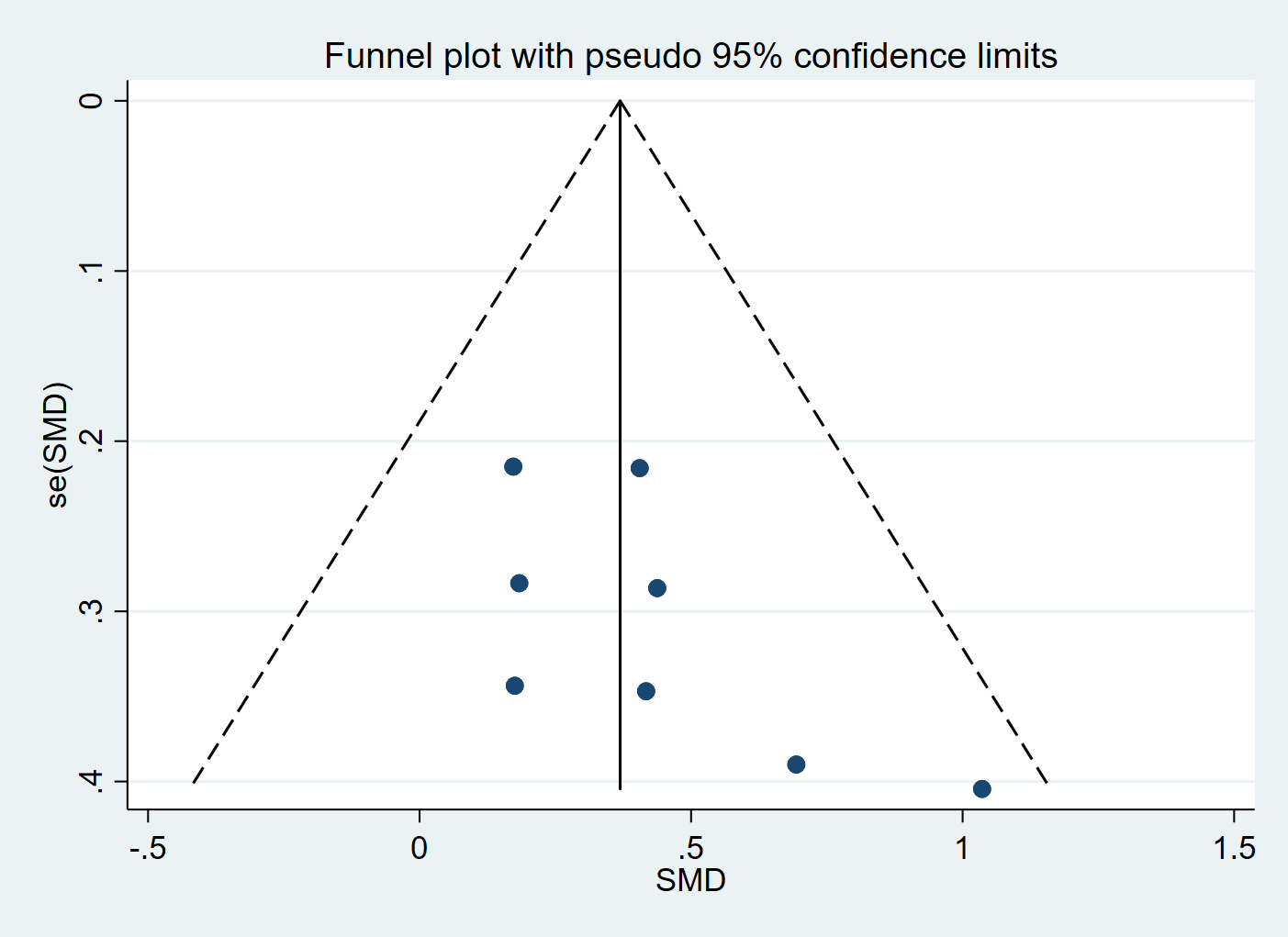

Supplement: Supplementary file 1 [file Datasheet1.zip › Supplementary files/S2 Funnel plot/Funnel plot Flexibility.tif]

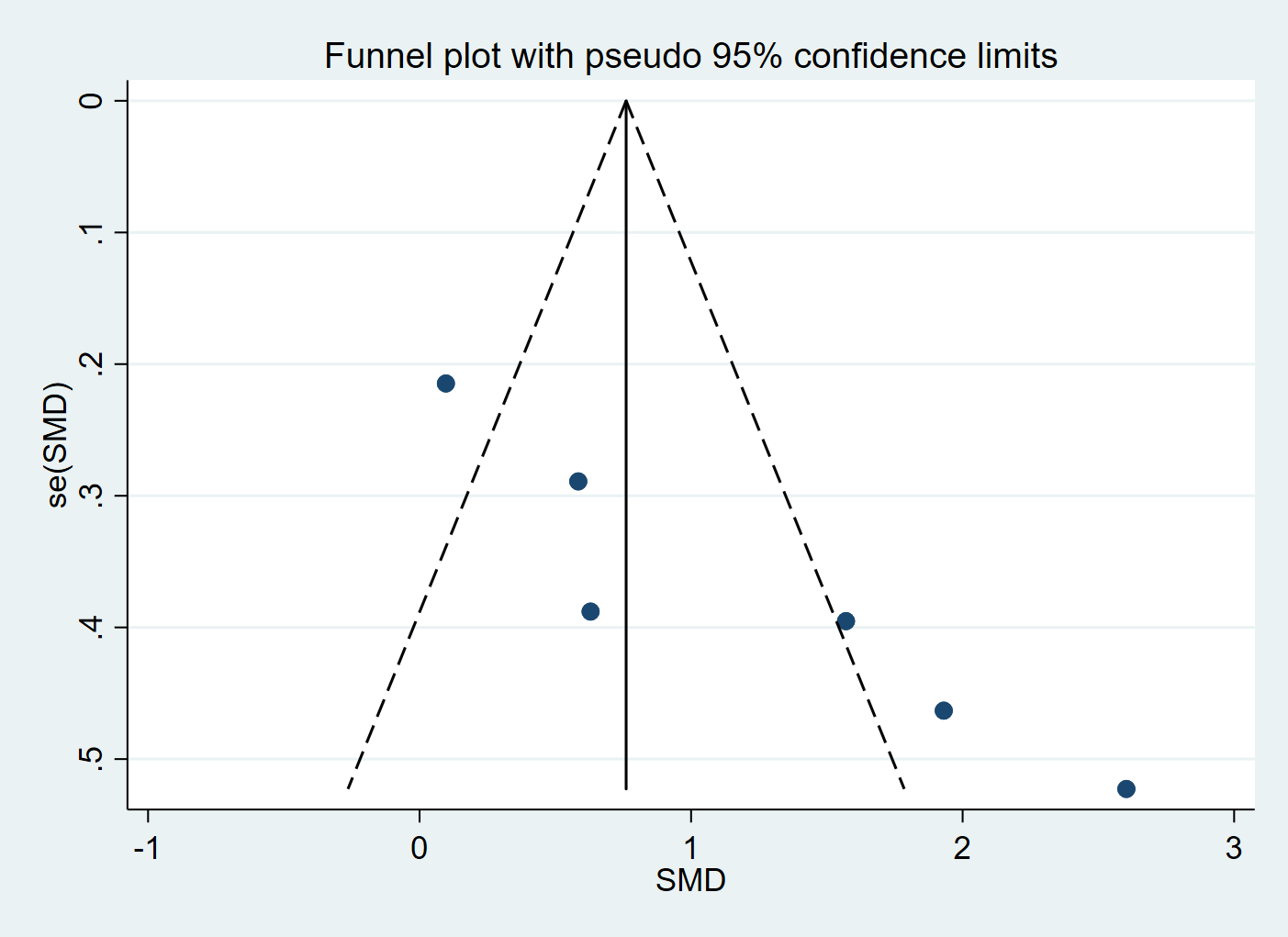

Supplement: Supplementary file 1 [file Datasheet1.zip › Supplementary files/S2 Funnel plot/Funnel plot Muscular Endurance.tif]

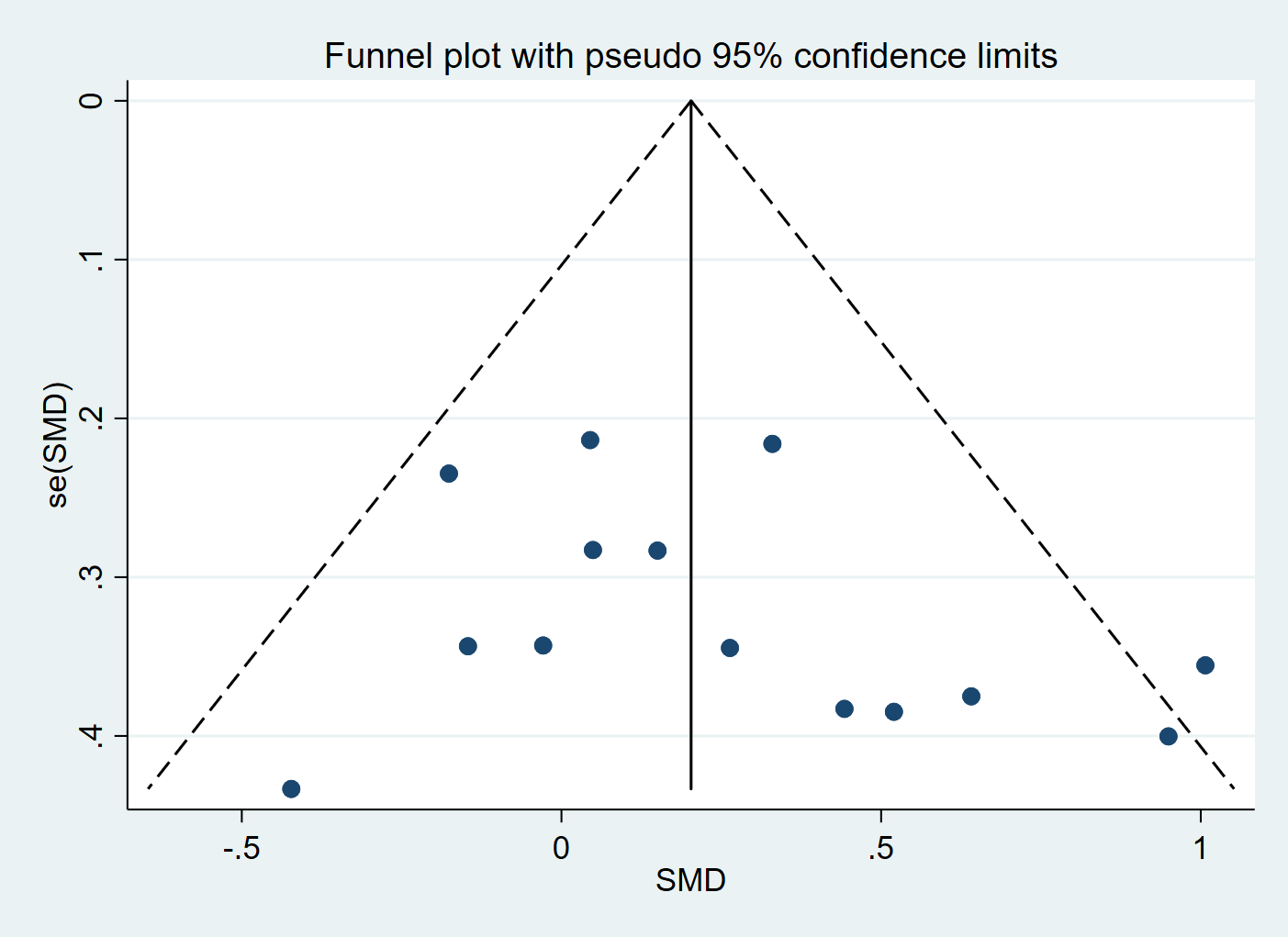

Supplement: Supplementary file 1 [file Datasheet1.zip › Supplementary files/S2 Funnel plot/Funnel plot Muscular Strength.tif]

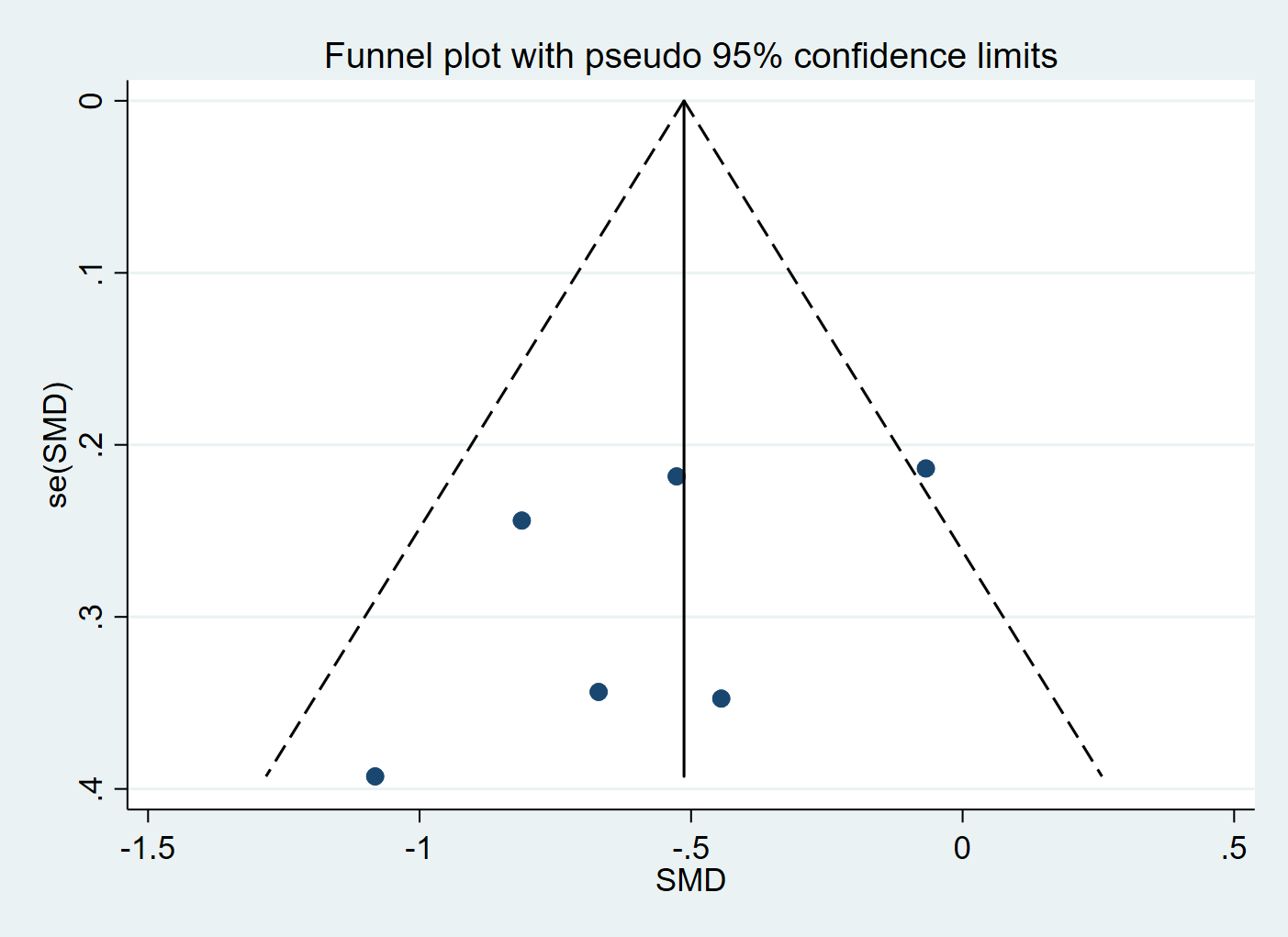

Supplement: Supplementary file 1 [file Datasheet1.zip › Supplementary files/S2 Funnel plot/Funnel plot Power.tif]

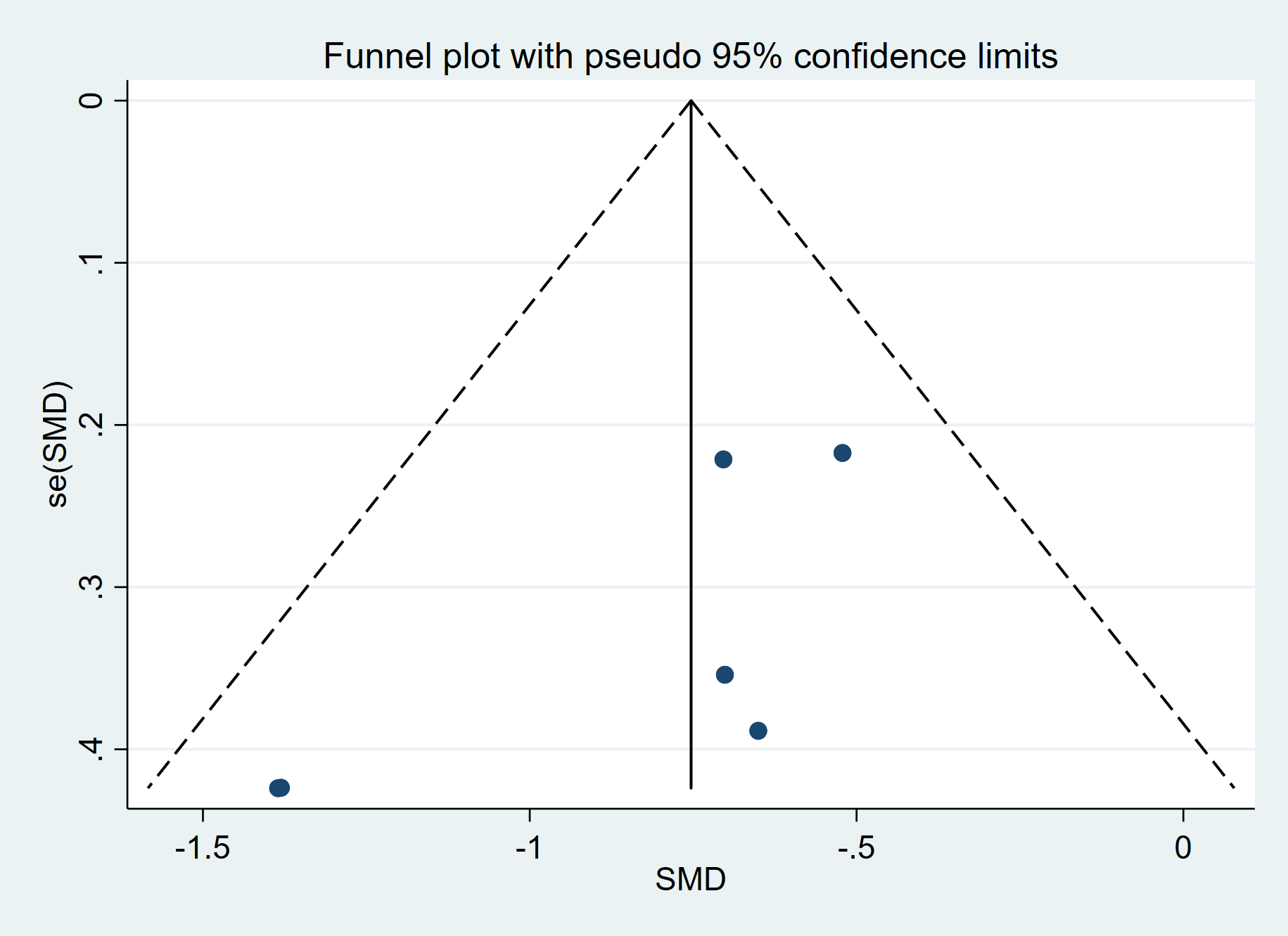

Supplement: Supplementary file 1 [file Datasheet1.zip › Supplementary files/S2 Funnel plot/funnel plot Agility.tif]

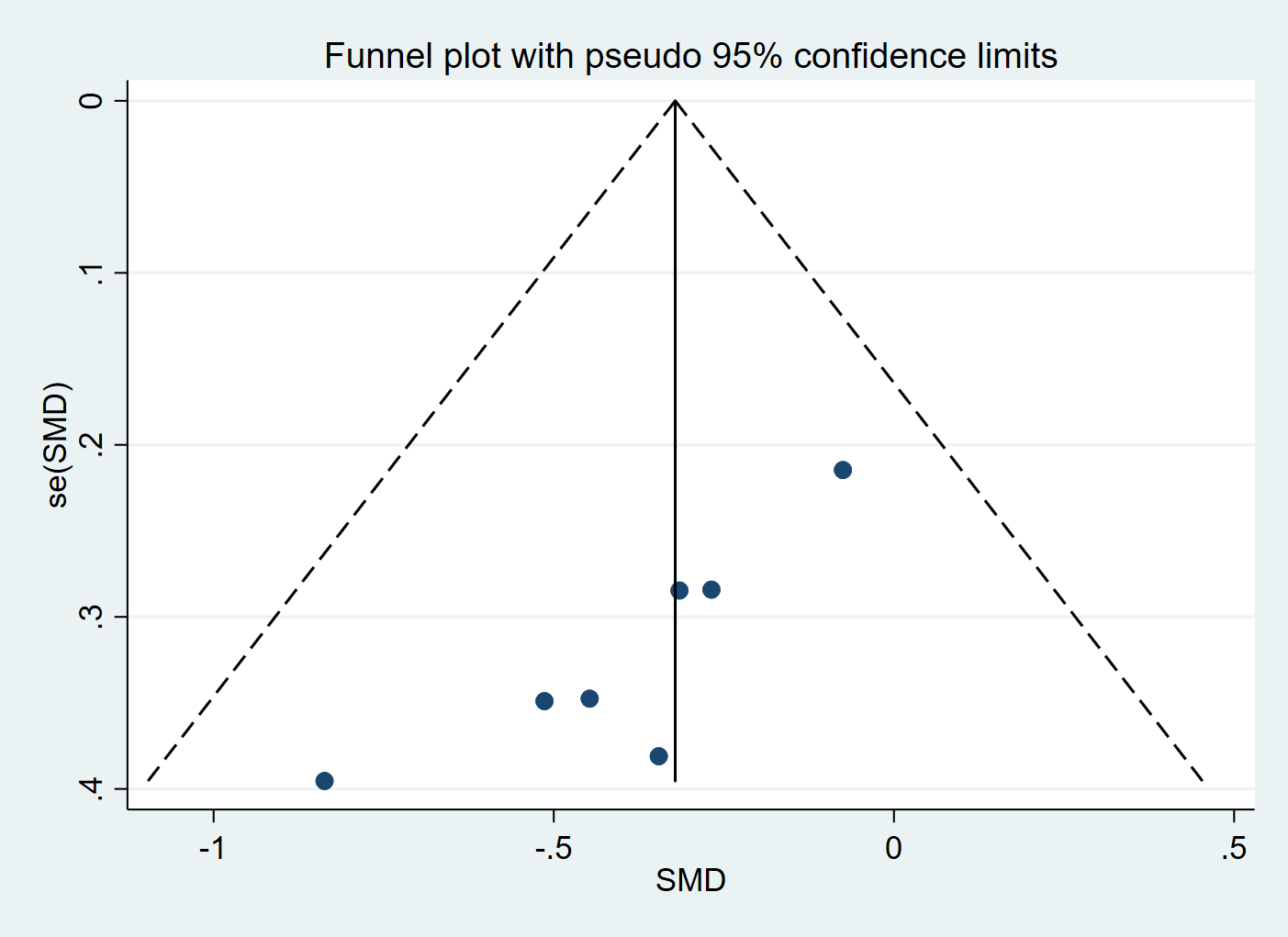

Supplement: Supplementary file 1 [file Datasheet1.zip › Supplementary files/S2 Funnel plot/funnel plot Speed.tif]
